# The Mode of SN38 Derivatives Interacting with Nicked DNA Mimics Biological Targeting of Topo I Poisons

**DOI:** 10.3390/ijms22147471

**Published:** 2021-07-12

**Authors:** Wojciech Bocian, Beata Naumczuk, Magdalena Urbanowicz, Jerzy Sitkowski, Anna Bierczyńska-Krzysik, Elżbieta Bednarek, Katarzyna Wiktorska, Małgorzata Milczarek, Lech Kozerski

**Affiliations:** 1 National Medicines Institute, 00-725 Warsaw, Poland; w.bocian@nil.gov.pl (W.B.); m.urbanowicz@nil.gov.pl (M.U.); j.sitkowski@nil.gov.pl (J.S.); e.bednarek@nil.gov.pl (E.B.); k.wiktorska@nil.gov.pl (K.W.); m.milczarek@nil.gov.pl (M.M.); l.kozerski@nil.gov.pl (L.K.); 2 Institute of Organic Chemistry, Polish Academy of Sciences, 01-224 Warsaw, Poland; 3 Łukasiewicz Industrial Chemistry Institute, 01-793 Warsaw, Poland; anna.krzysik@scopefluidics.com

**Keywords:** bioconjugate, camptothecin, DNA complexes, molecular modeling, ^1^H/^13^C NMR, DOSY, ESI–MS, MALDI–MS, Topo I poisons

## Abstract

The compounds 7-ethyl-9-(*N*-methylamino)methyl-10-hydroxycamptothecin (**2**) and 7-ethyl-9-(*N*-morpholino)methyl-10-hydroxycamptothecin (**3**) are potential topoisomerase I poisons. Moreover, they were shown to have favorable anti-neoplastic effects on several tumor cell lines. Due to these properties, the compounds are being considered for advancement to the preclinical development stage. To gain better insights into the molecular mechanism with the biological target, here, we conducted an investigation into their interactions with model nicked DNA (**1**) using different techniques. In this work, we observed the complexity of the mechanism of action of the compounds **2** and **3**, in addition to their decomposition products: compound **4** and SN38. Using DOSY experiments, evidence of the formation of strongly bonded molecular complexes of SN38 derivatives with DNA duplexes was provided. The molecular modeling based on cross-peaks from the NOESY spectrum also allowed us to assign the geometry of a molecular complex of DNA with compound **2**. Confirmation of the alkylation reaction of both compounds was obtained using MALDI–MS. Additionally, in the case of **3**, alkylation was confirmed in the recording of cross-peaks in the ^1^H/^13^C HSQC spectrum of ^13^C-enriched compound **3.** In this work, we showed that the studied compounds—parent compounds **2** and **3**, and their potential metabolite **4** and SN38—interact inside the nick of **1**, either forming the molecular complex or alkylating the DNA nitrogen bases. In order to confirm the influence of the studied compounds on the topoisomerase I relaxation activity of supercoiled DNA, the test was performed based upon the measurement of the fluorescence of DNA stain which can differentiate between supercoiled and relaxed DNA. The presented results confirmed that studied SN38 derivatives effectively block DNA relaxation mediated by Topo I, which means that they stop the machinery of Topo I activity.

## 1. Introduction

Targeted chemotherapy is a key element of personalized medicine that is now at the frontier of biomedical research. Covalent binding is a crucial factor in targeted chemotherapy because it damages the tumor DNA and substantially enhances the effectiveness of a medicine. Therefore, research aimed toward discovering drugs with site selectivity and strong binding to biological macromolecules has been a topic of longstanding interest due to the potential breadth and diversity of its application to basic, applied, and medicinal research.

Targeted chemotherapy requires a drug that binds strongly and in a site-specific manner to its biological target. In the case of derivatives of the camptothecin family acting as the topoisomerase I (Topo I) poisons [1], this site is defined as a nick in the DNA of the inhibitor/DNA/Topo I ternary complex [2,3].

The inhibition of topoisomerase I is, thus, an important strategy in fighting cancer using chemotherapy. A number of effective bioorganic strategies are used to achieve this goal, such as the noncovalent binding of the poison at the site of degradation, [4,5] cross-linking using binding metals, [6] DNA alkylation [7,8,9,10] or photochemical DNA damage [11].

**Scheme 1:**
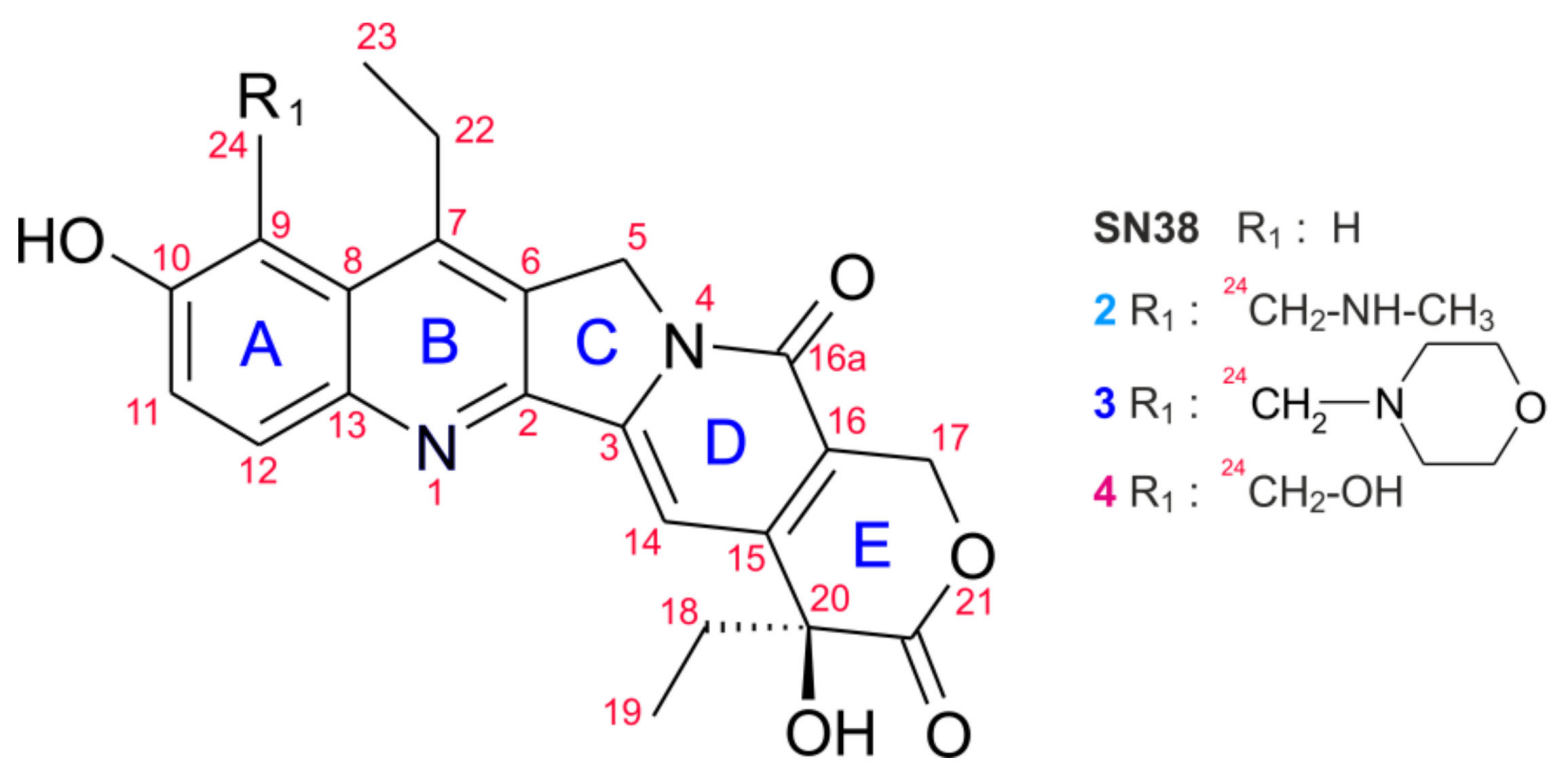
The structure of SN38 and derivatives **2–4**.

**Scheme 2:**
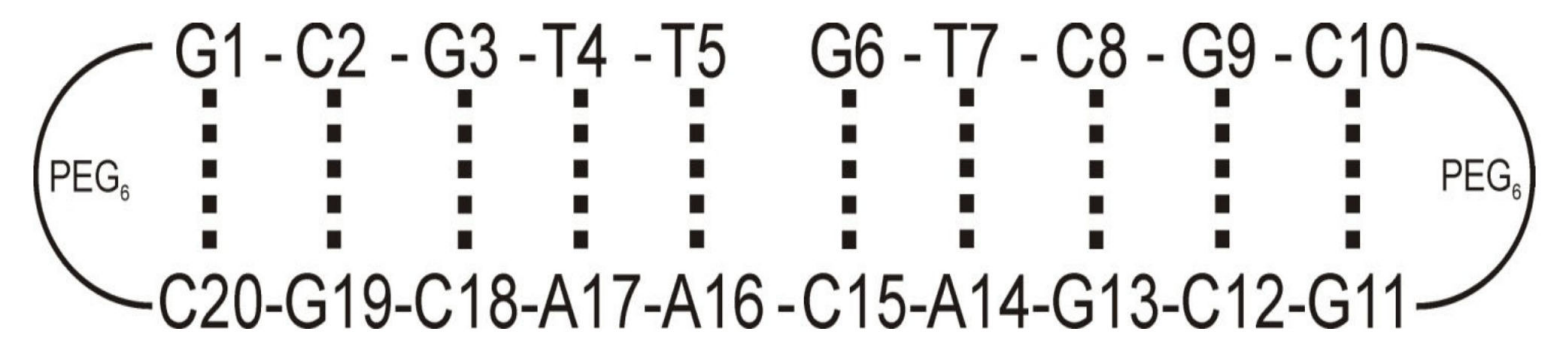
Schematic representation of the biological target in the nicked DNA duplex in a model decamer with a PEG(6) tether at both ends of the duplex (Supplementary Materials Figure S1). The GC base pair on the right side of the nick (GC side) in nicked DNA portrays the nick environment in wild-type DNA.

During the course of earlier research on camptothecin derivatives, we presented evidence for the formation of a molecular complex with the camptothecin core of topotecan (Hycamtin™), which occurred via selective interaction with the ultimate GC base pair in a natural DNA oligomer [12] and inside the nick of the model nicked DNA decamer [5,13]. Further studies [14] led us to disclose new SN38 derivatives that spontaneously alkylate DNA oligomers [15,16,17]. Covalent binding was confirmed in model 2′-deoxyguanosine (dG) [18], 2′-deoxycitidine (dC) [19], and 2′-deoxyadenosine (dA) [20] nucleosides. The GC base pair is considered to be a prerequisite presence in a nicked site of DNA for Topo I inhibition [21]. These results prompted us investigate the present compounds (Scheme 1) with the hope of acquiring more information on the mechanism of their interaction with model nicked DNA (Scheme 2). The possibility of the alkylation of the nitrogen bases inside the nick by potential Topo I poisons would result in pharmaceutics safe for a patient, a priority feature in oncological therapy, and devoid of side effects. This is especially important in view of the fact that the camptothecin derivative Irinotecan used in colorectal cancer therapy, considered by WHO as ‘an essential medicine’, metabolizes to compound SN38 which may result in lethal diarrhea.

The clear explanation for the above reasoning is found in reviews of Pommier et al. [22] who developed the concept of an ‘interfacial inhibitor’ role in the machinery of Topo I activity poisoning, explained on examples of experimental results and confirmed by metadynamic in silico modeling [23]. The central module of action of machinery is the interface of a ternary complex Topo I/DNA/Inhibitor. Most of the interfacial inhibitors are primarily kinetic inhibitors forming molecular complexes with ‘on–off’ rate timing and therefore all contacts between inhibitor and both enzyme and DNA are essential to keep the complex stable and reduce the ‘off-rate’. In the present contribution, the camptothecin derivatives are shown to alkylate the nitrogen bases on the face of a nick in DNA and they totally reduce the ‘off-rate’, stopping the machinery of Topo I activity. In other words, they act as a tool braking on the cogs in a working gear, an activity we wish to highlight in our contribution.

No reports have been published regarding compounds from the camptothecin group that are able to alkylate DNA without the assistance of chemistry or photochemistry, and instead, react spontaneously and therefore have potential for use as medicines in vivo. The mechanism of favorable cytotoxic activity of these compounds [17] has yet to be thoroughly investigated. Preliminary results of experiments on derivatives with DNA oligomers disclosed that, in addition to the alkylation products in a reaction mixture, compounds SN38 and 7-ethyl-9-hydroxymethyl-10-hydroxycamptothecin (**4**) were found. The formation of these side products influences the yield of desired alkylation and modulates the biological activity of the parent compound under in vitro physiological conditions. This situation is the basis for a discussion regarding the involved mechanism leading to the observation of the biological activity of compounds at the cellular level based on physicochemical properties, such as the half-life time of chemical stability, strength of intercalation, and kinetics of the reaction with oligonucleotides. Explanation will require the consideration of the structural properties that determine the biological activity through mechanisms such as alkylation via a quinone methide intermediate, direct nucleophilic substitution on the 9-C–**Cα** carbon atom, and retro-Mannich reaction on the 9-C atom. This discussion will hopefully allow for a better understanding of how camptothecin derivatives can poison cancer cells better than other Topo I inhibitors. Therefore, in this study, we attempted to experimentally address these issues.

Previously, we designed the nicked DNA decamer [13] of structure **1** (Scheme 2), which has the GC base pair as a face in the nick and is able to interact with potential Topo I poisons.

It was previously shown [17] that studied compounds (Scheme 1) (Figures S2 and S3) have preferable anti-neoplastic effects on several cancer cells, and the derivatives **3** and **4** are nontoxic to normal cells. These properties prompted us to attempt to establish their pharmacological role at a molecular level as being Topo I poisons, as part of a process of increased understanding toward advancing the new camptothecins to the early phases of clinical studies. Accordingly, in this paper, we present NMR-derived evidence of the site-selective interaction of the SN38 derivatives 7-ethyl-9-(*N*-methylamino)methyl-10-hydroxycamptothecin hydrochloride (**2**) and 7-ethyl-9-(*N*-morpholino)methyl-10-hydroxycamptothecin hydrochloride (**3**) with the nick in the model decamer **1**. We deliberately chose the two derivatives with the secondary and tertiary amine in a substituent at 9-C to determine the eventual differences in binding and reactivity.

We report the results alternatively for compounds **2** and **3**, providing all complementary important information in the Supplementary Materials.

The preliminary results showed that the derivatives **2** and **3** react slowly in solution in the presence of DNA oligomers. Therefore, it was decided that the geometry of the molecular complex be characterized at an early stage of reaction and the products analyzed after the reaction was completed.

The kinetics of the interaction were followed by monitoring the ^13^C resonances in the 24-^13^C-enriched carbon atom in **3**. Molecular modeling, using NOESY-derived cross-peaks, allowed the presentation of the geometry of the inclusion complexes in the nick of **1**. The DOSY experiment on the final reaction product in the solution and MALDI–MS spectra of solid products allowed the confirmation of the existence of a site-selective alkylation of a base in the nick, in addition to the strong inclusion of molecular complexes with parent compounds and their hydrolysis product, compound **4**.

## 2. Results and Discussion

### 2.1. DOSY Experiments of Molecular Complexes

Diffusion ordered spectroscopy (DOSY) is a convenient NMR experiment for the quantitative analysis of the affinity of interacting molecules in solution [24,25]. It is based on measuring the diffusion coefficients Di × 10^−10^ (m^2^ s^−1^) of both free and complexed compounds. The small ligands **2** or **3** will have much smaller diffusion coefficients upon binding interaction with the larger host molecule **1** compared with the diffusion coefficients when they are free in a solution. The methodology for moving from diffusion coefficients to affinity constants is given in the Materials and Methods. The present results suggest (Table 1) that the binding is very strong. The diffusion coefficient D_i_ for neat decamer **1** in the cited conditions is 1.13 × 10^−10^ (m^2^ s^−1^) [5] and 3.07 × 10^−10^ (m^2^s^−1^) for derivative **2.** Taking into account that the derivative **2** is in a molar excess, the observed diffusion coefficient Di 1.47 × 10^−10^ (m^2^ s^−1^) is the mean value of a free and bound **2.** Nevertheless, this experiment shows that both interacting molecules form a strong molecular complex. Table 1 shows the data used to calculate the binding constant of a complex. The data in Table 1 show that almost all of compound **1** is involved in a complex with guest **2.** The DOSY experiments results for ligands **2** and **3** under similar conditions are presented in the Supplementary Materials (Figures S4,S5 and Figure S6a,b, respectively).

Therefore, a NOESY experiment was planned to gain insights into the geometry of the complex. Compound **2** was chosen for this experiment because it is easier to monitor the fate of the parent compound during the study period using a sharp N–CH_3_ signal (Figures S2 and S7).

### 2.2. NOESY Experiment of the Molecular C Complex 

The NOESY and TOCSY experiments are two-dimensional NMR techniques commonly used to study macromolecules such as proteins and DNA. TOCSY is an experiment in which we observe cross-peaks on spectra not only for directly coupled nuclei, but also between nuclei connected by a chain of couplings. This makes the technique very useful for the correct assignments of protons in individual units of a DNA sequence. The NOESY experiments are used to establish correlations between nuclei that are physically close to each other (correlation through the space within approximately 5 Å), whether or not there is a bond between them. The NOESY technique is typically used to determine the DNA sequence. Since we can observe the correlation peaks between the two interacting molecules on the NOESY spectra, the technique was also commonly used to study DNA molecular complexes with ligands.

The NOESY and TOCSY spectra were used to assign the protons’ chemical shifts of decamer **1** and compound **2** in complex at 1:2 ratio (Table S1 and Table 2), including the assignment of all the found intermolecular cross-peaks. Table 3 presents the changes induced in chemical shifts of neat decamer **1** [13] by interaction with **2**. They are confined to the two base pairs flanking the nick because negligible changes were observed in more distant base pairs (Table 3 and Table S1). Table 2 also shows changes in chemical shifts induced in protons in compound **2** by interaction with decamer **1** in a complex.

**Figure 1:**
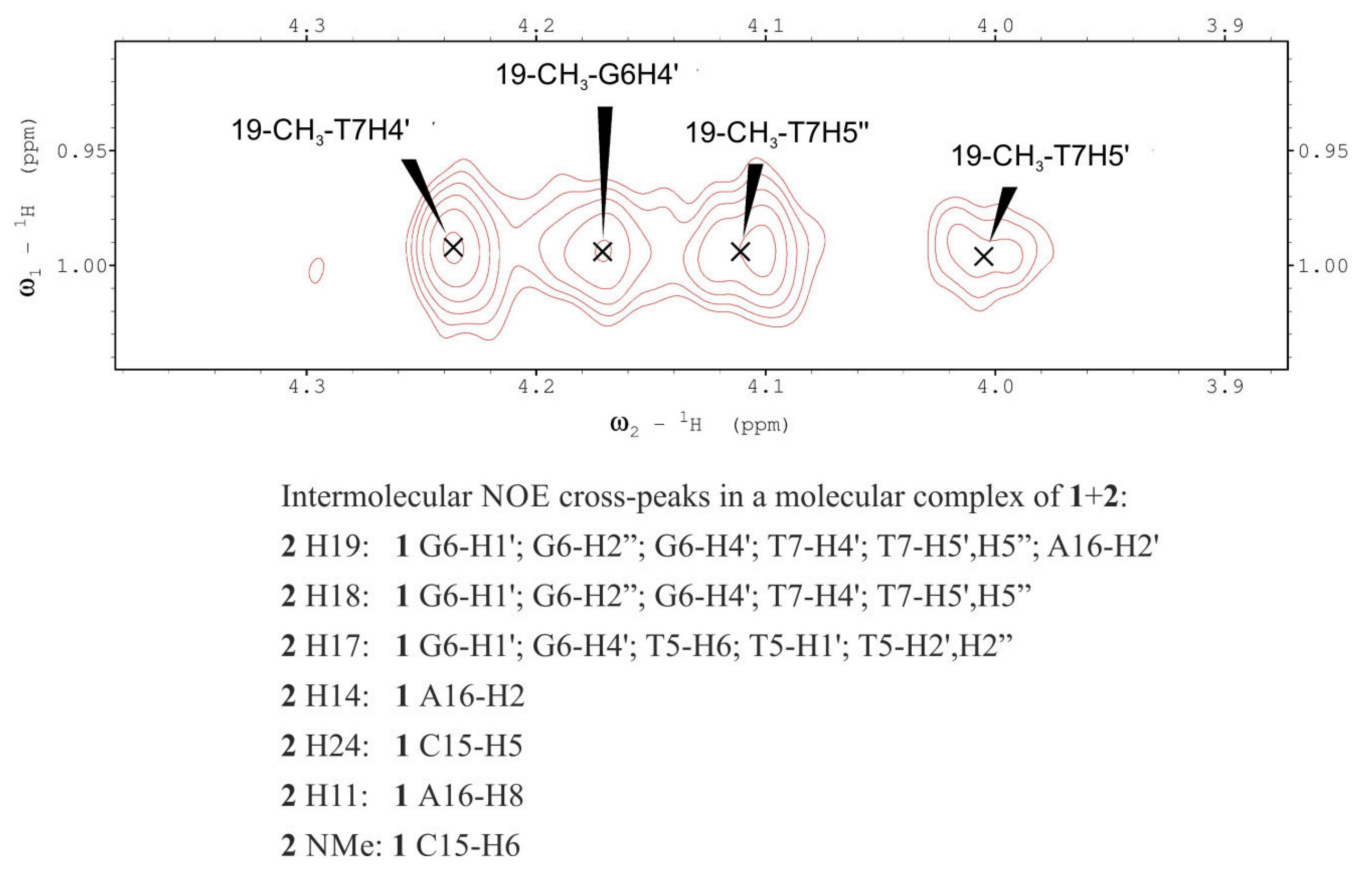
The list of NOE cross-peaks found for molecular complex **1** + **2** and the part of the NOESY spectrum representing the intermolecular cross-peaks between 19-CH_3_ group in **2** and DNA bases in the nick of **1** (Figures S8 and S8a). For the NOESY and intermolecular cross-peaks in a complex **1** + **3**, see Figures S9 and S10.

The data presented in Table 2 and Table 3 show that, in both interacting molecules, most protons undergo low frequency shifts, which indicates the stacking of the aromatic core of derivative **2** and nitrogen bases in **1** (see Section 2.3*).* Strong interaction thus allows the intermolecular cross-peaks shown in Figure 1 to be observed.

The integrated volumes of all intermolecular NOE cross-peaks are cited in Table 4. These are given as a percentage of an intramolecular cross-peak volume between isolated pairs of spins of protons 11-H and 12-H in compound **2**.

Based on the presented experimental data, several remarks can be made concerning the origin and geometry of a complex. From the presented complementary data of DOSY and NOESY, the conclusion can be drawn that interacting molecules **1** and **2** form a complex, which is in fast exchange at a chemical shift time scale at the NMR frequency used (500 MHz). The single spectra of both interacting species are observed. The correlation time of a molecular complex, its strong binding, and its apparent high population in an equilibrium at the NMR time scale and temperature used in the experiment allow the observation of dipolar contacts in a complex in the form of cross-peaks of intensity comparable to the intensity of intramolecular cross-peaks.

**Figure 2:**
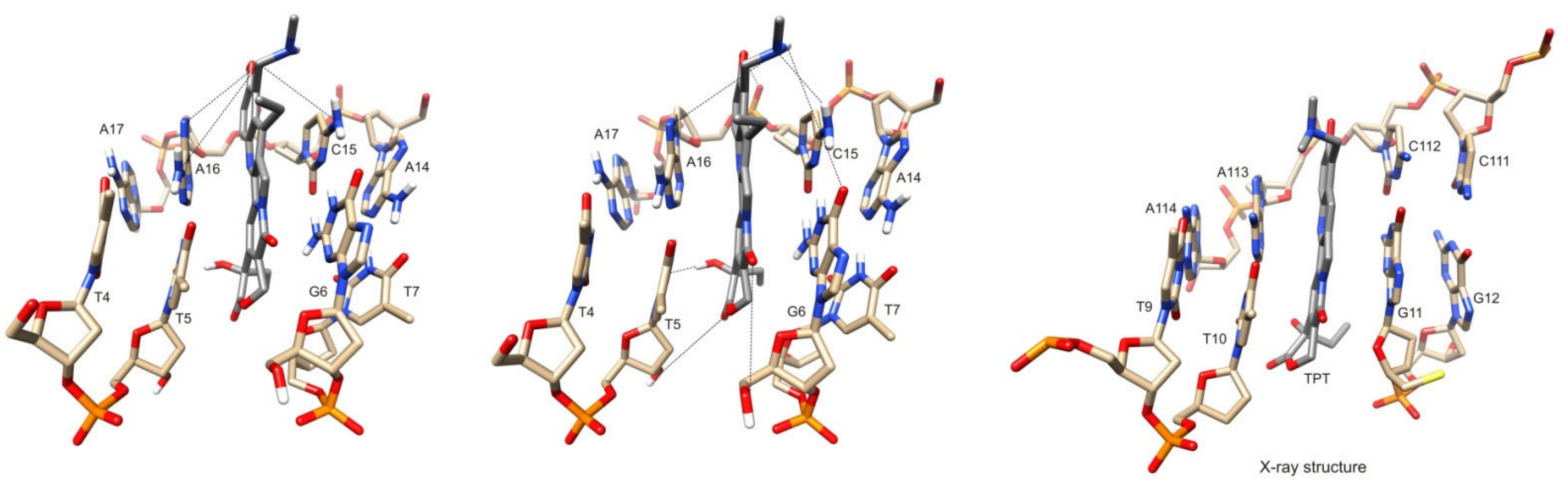
Molecular view of the most probable geometry of complex **1** + **2.** Structure 1 is from MD trajectories (Figure S11*).* The N–CH_3_ group is pointing towards a major groove. The left panel shows internuclear contacts for possible hybrid formation. The center panel shows possible hydrogen bond network stabilizing the structure (see Figure S13 and Table S4 for internuclear distances and assignments). For clarity, only two base pairs flanking the nick are shown. Right panel shows the fragment from X-ray complex of nicked 20-mer nicked DNA with topotecan (RCSB PDB:1K4T). [26].

Both solutes are in exchange but strongly interacting, as indicated by DOSY. Relatively large chemical shift changes to lower frequencies, upon complex formation, also point to a mutual strong influence, suggesting stacking as an interaction mode. This reasoning is also true for the rigid positioning of a camptothecin core of topotecan entrapped in a nick of a crystal structure of a complex DNA/TPT, as shown in Figure 2.

The cross-peaks are nearly exclusively found in base pairs flanking the nick. This indicates that the guest molecule is placed in the nick. Furthermore, the individual cross-peaks indicate that ring A of compound **2** interacts with bases C15 and A16 in an unbroken strand of a duplex, whereas ring E is facing the T5 and G6 bases in a broken strand. This hints strongly at the geometry of a guest in the nick, as shown in Figure 2. It was therefore decided to qualitatively use these cross-peak volumes in a protocol of molecular modeling to confirm the above indications and choose one of the four possible geometries of stacking for the guest molecule in the nick of decamer duplex **1.** The alternative protocol of quantitatively using cross-peak volumes is not possible in the present case due to experimental constraints in calculating the complex structure. The complexity of the mechanism of action prevents the construction of the buildup of small cross-peak volumes using mixing time in the NOESY experiment in a range of 50–200 µsec.

### 2.3. Molecular Modeling

The SN38 derivative was manually docked to the model structure of the nicked DNA receptor previously established by authors [13] in all four possible stacking orientations. The two starting orientations of the bulky substituents in **2** (9-CH_2_–NH–CH_3_ and 7-CH_2_–CH_3_) relative to the remainder of the camptothecin core were taken into consideration, resulting in a total of eight systems, which were then each subjected to 500 ns molecular dynamics (MD) calculations. The trajectories—which were doubled due to the starting orientation of the substituents—were combined into one, resulting in four distinct structures (Table S3). Table 4 shows the result of an analysis of the ensemble of conformations for each cluster, representing four structures of initial stacking orientation of **2** in the nick (Figures S11–S13; Tables S3 and S4). The first two columns define the interacting protons that give rise to a cross-peak, whereas column 3 indicates the distance between them, reflected by a cross-peak volume, which is calibrated vs. the distance between the 11-H and 12-H protons of **2**. In an ensemble of conformations defining the four trajectories, the distance in each conformation between the given pair of protons was statistically evaluated according to five defined conditions (in Å), and the percentage of conformations meeting this requirement is shown in Table 4. Inspecting Table 4 clearly points to the structure of 1 (Figure 2) as the one which meets the requirements in a high percentage. It is worth noting that a high percentage of conformations meet the requirement of <4 Å, a distance which can give reliably measurable intermolecular NOE effects.

**Figure 3:**
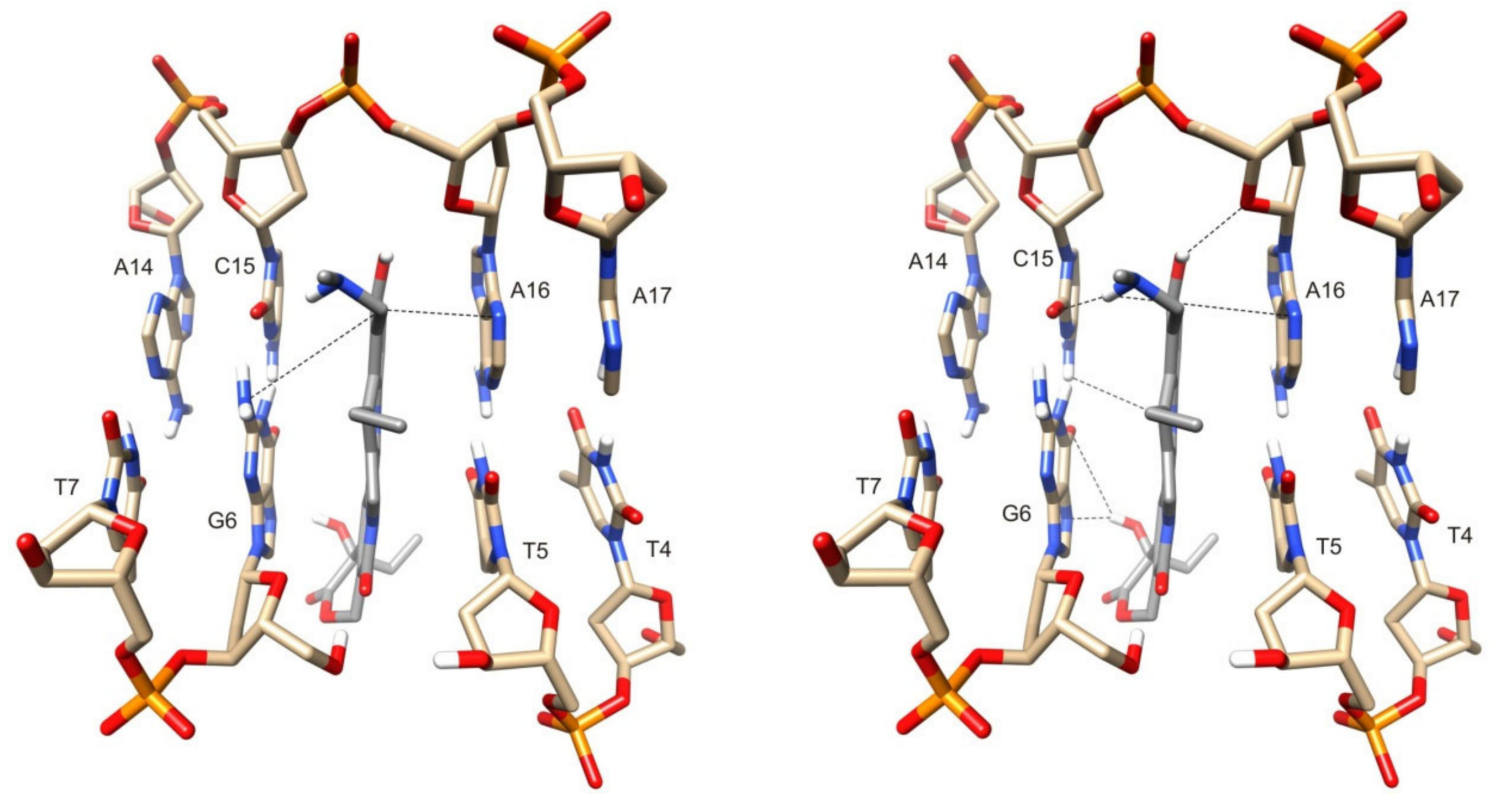
Molecular view of the second probable geometry of a complex **1** + **2.** Structure 3 from MD trajectories (Figure S12). The molecule of **2** is turned over by 180° with respect to structure 1. The N–CH_3_ group is pointing into a minor groove. The left panel shows internuclear contact for possible hybrid formation. The right panel shows possible hydrogen bonds stabilizing the structure (see Figure S13 and Table S4 for internuclear distances and assignments).

Furthermore, some statistical confirmations were found for structure 3, which is also characterized by facing the ring A of **2** with the unbroken strand, but the guest is turned over by 180° inside the nick along its long axis (Figure 3). Hence, the presence of cross-peaks is observed between the T5 base in a broken strand and 17-H of **2** (Figure 3). Further evidence of this is the structure in Figure 3. This geometry of a guest inside the nick justifies the absence of cross-peaks between 19-CH_3_ and ribose protons in G6, T7 in the molecular modeling of structure 3 (Table 4). This methyl group is further from the ribose rings of G6 and T7 than in structure 1 because it is on the opposite side of a camptothecin ring E.

The calculated averaged energies for MD trajectories for the investigated structures are given in Table S3. The differences between these are less than the accuracy of the calculations. Therefore, in this work, we place the main emphasis on the comparison of the calculated geometric parameters (dipolar contacts represented by cross-peaks due to the stacking of the aromatic rings of interacting species and intermolecular hydrogen bonds). Nevertheless, the energy analysis also confirms the preference for structures 3 and 1.

The distances between the given protons represented by cross-peaks in Table 4 fulfill most of the imposed conditions. Table 4 reports the statistical analysis of dipolar contacts in structures 1 and 3, and Table S4 reports the analysis of hydrogen bonds in ensembles 1–4. The dipolar contacts favor structure 1, but hydrogen bonding is favored in structure 3, with a higher percentage of existing hydrogen bonds compared with structure 1 (Table S4). Both phenomena (stacking and hydrogen bonding) contribute energy to the complex formation and therefore structures 1 and 3 were considered to best represent the geometry of a complex. Moreover, the geometry of structure 1 is also very similar to that seen in the X-ray structures of ternary complex Topo I/DNA/TPT [26] as shown in Figure 2. It is clearly seen that in both cases, the positioning of a camptothecin core is the same; with ring A facing A, C bases and ring E facing the T, G bases in a scissile strand. Additionally, in both cases, the substituents on the 9-C carbon atom, N-CH_3_ and N-(CH_3_)_2_ groups point towards a major groove. The camptothecin core is trapped in a nick mimicking stacking mode as if it were an additional base pair. These results are in agreement with the earlier molecular modeling of docking camptothecin core into a ternary cleavable complex. [27,28]

Both computed structures in Figure 2 and Figure 3 can be plausibly confirmed by the experimental results given in Table 2 and Table 3 and the observed cross-peaks. First, the observed changes of chemical shifts on DNA protons, upon complex formation, are almost entirely confined to aromatic bases flanking the nick from both sides. Second, the low frequency shifts induced on protons in **2** allow clear suggestions concerning the positioning of a camptothecin core inside the nick. The protons around ring A, namely, 11-H, 24-H, and N–CH_3_, show cross-peaks with the aromatic protons of C15 and A16, and therefore experience large low-frequency shifts being in the shielding cones of these aromatic bases from both sides. By comparison, the protons around ring E, namely 17-H, 18-H, and 19-H, have cross-peaks with the aromatic protons of G6 in a broken strand. This increases confidence in the identification of the nitrogen bases inside the nick of derivative **2** as possible sites of alkylation (Table 5). It can be supposed that the geometry of both structures 1 and 3 make alkylation of nucleophilic bases possible in the nick (Table 5). In structure 1, atom 24-C of a transient methide is facing the NH_2_ group of cytosine C15 or adenosine A16 and the N7 nitrogen atom of the adenosine. This course of a kinetic alkylation of N7 nitrogen atom was recently discussed in the reaction of compounds **2** and **3** with model 2′-deoxyadenosine [20]. In addition, structures 1 and 3 are stabilized by a network of hydrogen bonding from both ends of a camptothecin core (Figure S13, Table S4). In structure 3, the alkylation of the NH_2_ group of guanosine G6 is possible, and finds precedence in a recently published result of the alkylation of model 2′-deoxyguanosine [18]. The alkylation of the N3 nitrogen atom in adenine A16 is also indicated, as shown in a projection of structure 3, although it is less probable in view of earlier results [20].

### 2.4. The Reaction Products Analysis

As the reaction solutions contain an excess of compound **2**, which influences the value of the diffusion coefficient, D_i_, it was decided to remove it to establish the condition that enables the recording of the interaction in a 1:1 molar ratio of components. After filtering the reaction solution through a membrane to remove the unbound **2** and 7-ethyl-9-hydroxymetyl-10-hydroxycamptothecin, **4**, the mother liquor (ML), was further incubated at room temperature for several weeks and the ratio of T7-CH_3_ and 19-CH_3_ was monitored by ^1^H NMR integration. The sample stabilized at the 1:1 ratio of components **1** and **2.** The DOSY experiment in Figure S5 shows equal diffusion coefficients for both reactants (D_i_, 1.0 × 10^−10^ (m^2^ s^−1^)) in solution, indicating very strong binding in a molecular complex. In addition, the ^1^H NMR of the mother liquor (ML) (Figure S7) shows the same integration of N–CH_3_,19-CH_3_ of **2**, and T7-CH_3_ of **1**, indicating that parent compound **2** is a major component in the molecular complex with **1.**

**Figure 4:**
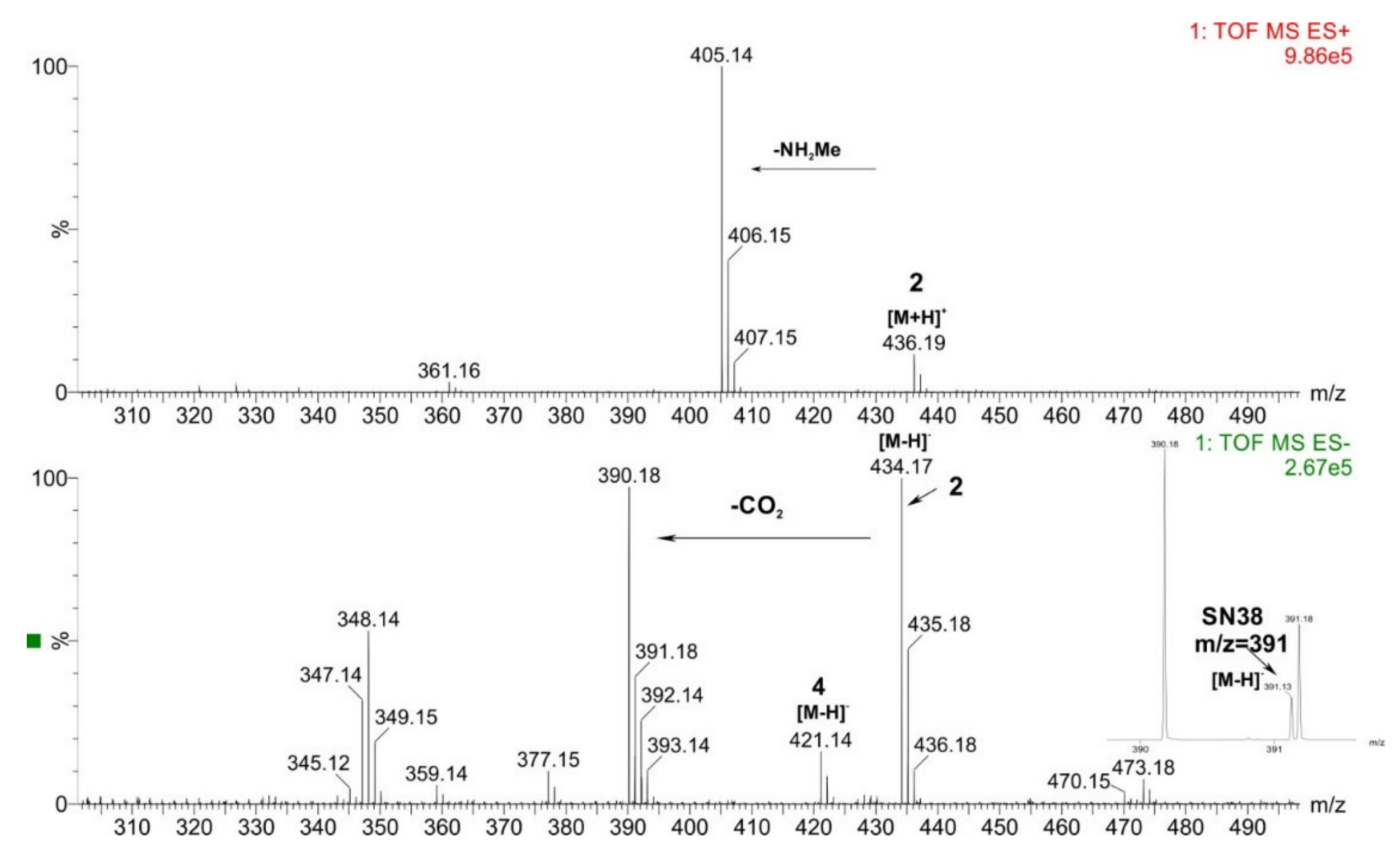
The ESI–MS spectrum (in the *m/z* range 300–500) of positive (upper trace) and negative ions (lower trace) of mother liquor of reaction **1** with **2**.

**Scheme 3:**
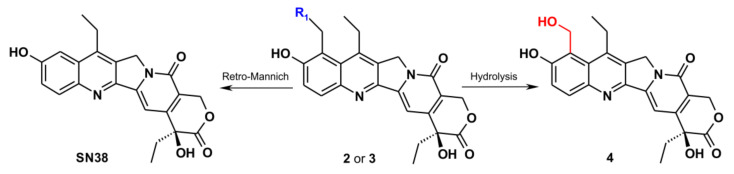
Metabolites of parent compounds.

**Figure 5:**
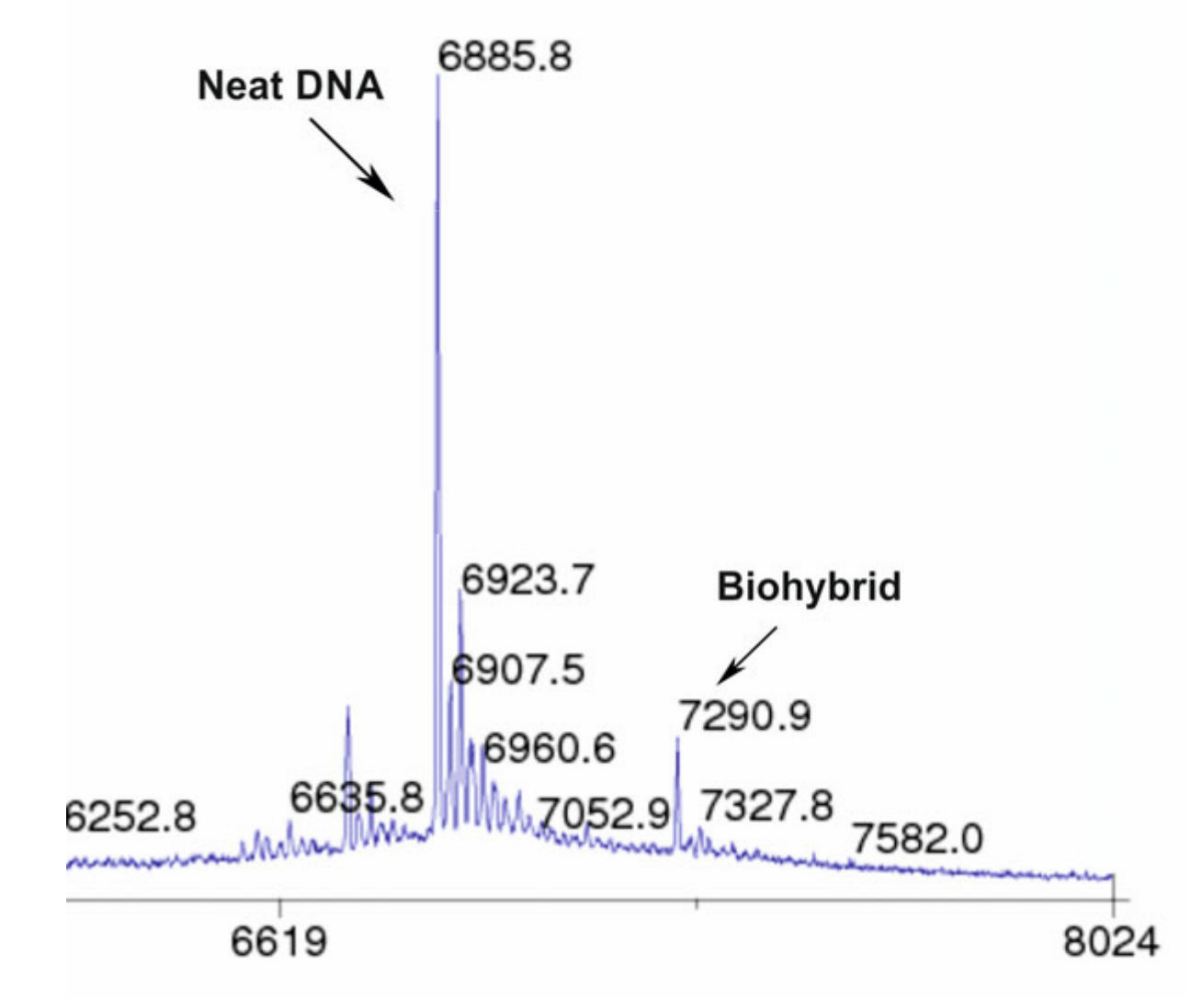
The part of the MALDI–MS spectrum of negative ions showing the mother peak for the neat parent nicked decamer, at *m*/*z* = 6885.8 ([M − H]^−^), and a peak of the biohybrid of **2** with decamer **1** at *m*/*z* = 7290.9. The difference in masses M = 405 is due to transient intermediate *o*-methylene quinone formed from parent compound **2** or **3** [14].

These indications are confirmed in the ESI–MS spectrum of the mother liquor shown in Figure 4, which presents the *m*/*z* range 300–500 of the ESI–MS spectrum of positive and negative ions. It shows mother peaks for parent compound **2** (*m*/*z* = 436, [M + H]^+^) and its hydrolysis product **4** (*m*/*z* = 421, [M − H]^−^) as guest ligands of a broken molecular complex with **1**. Interestingly, and also importantly, there is only a minor intensity peak due to SN38 (*m*/*z* = 391, [M − H]^−^) in the negative ion spectrum (product of the retro-Mannich reaction, Scheme 3). In agreement with this, the MALDI–MS spectrum of lyophilized mother liquor in Figure 5 (Figure S14) shows free compound **1** (*m*/*z* = 6885.8) as a host molecule of a broken molecular complex and alkylated product (*m*/*z* = 7290.9).

The above information from DOSY, NOESY, MD, MALDI–MS, and ESI–MS spectra allows the conclusion to be drawn that compound **2**, either in the form of a molecular complex or alkylated biohybrid, is bound inside the nick of a model DNA, mimicking the target for Topo I poisons regardless of the internal geometry of molecular complexes depicted as structures 1 and 3 from MD (Figure 2 and Figure 3). In both cases, the prerequisite conditions required for Topo I poisons, i.e., binding in a nick, are fulfilled.

**Figure 6:**
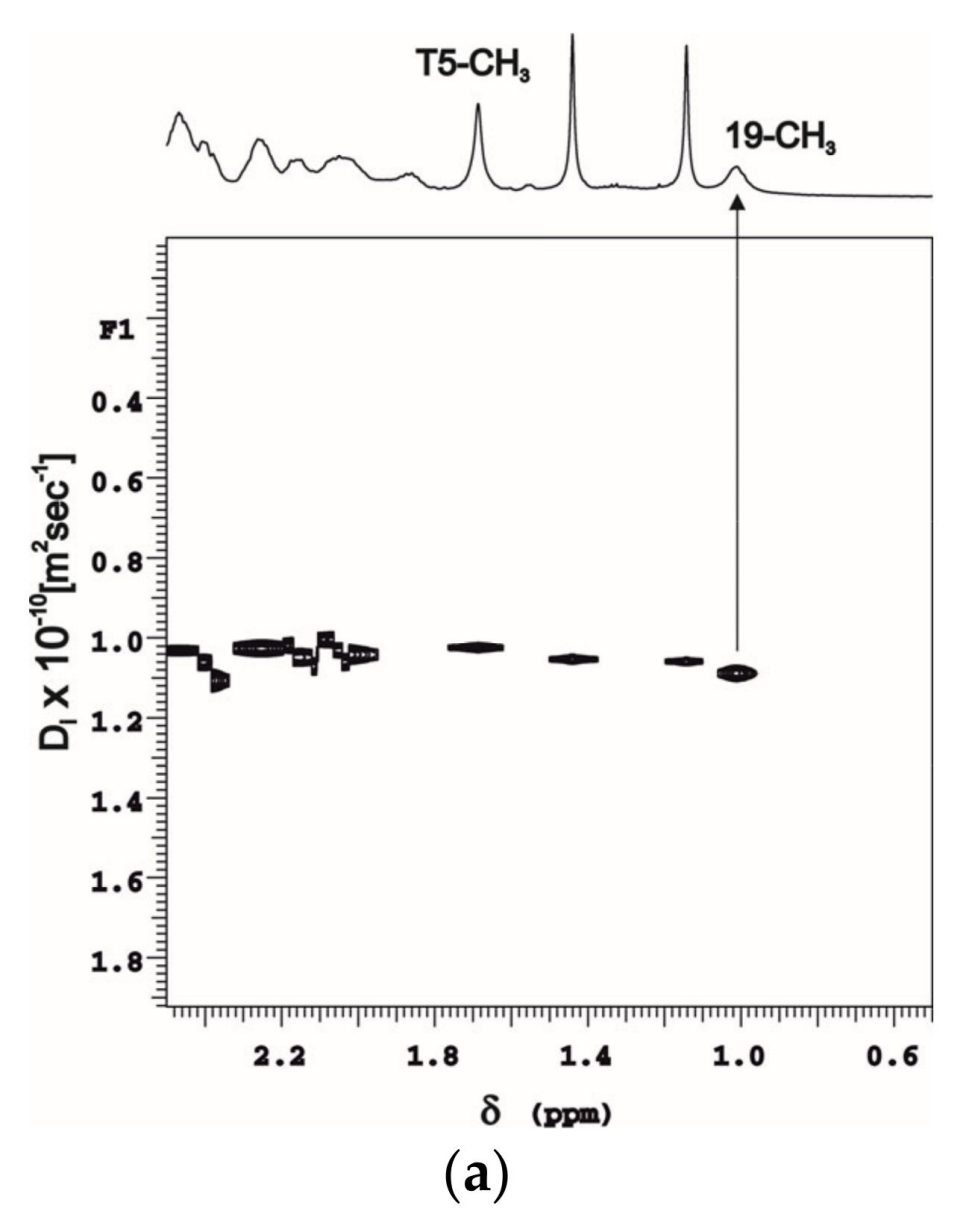

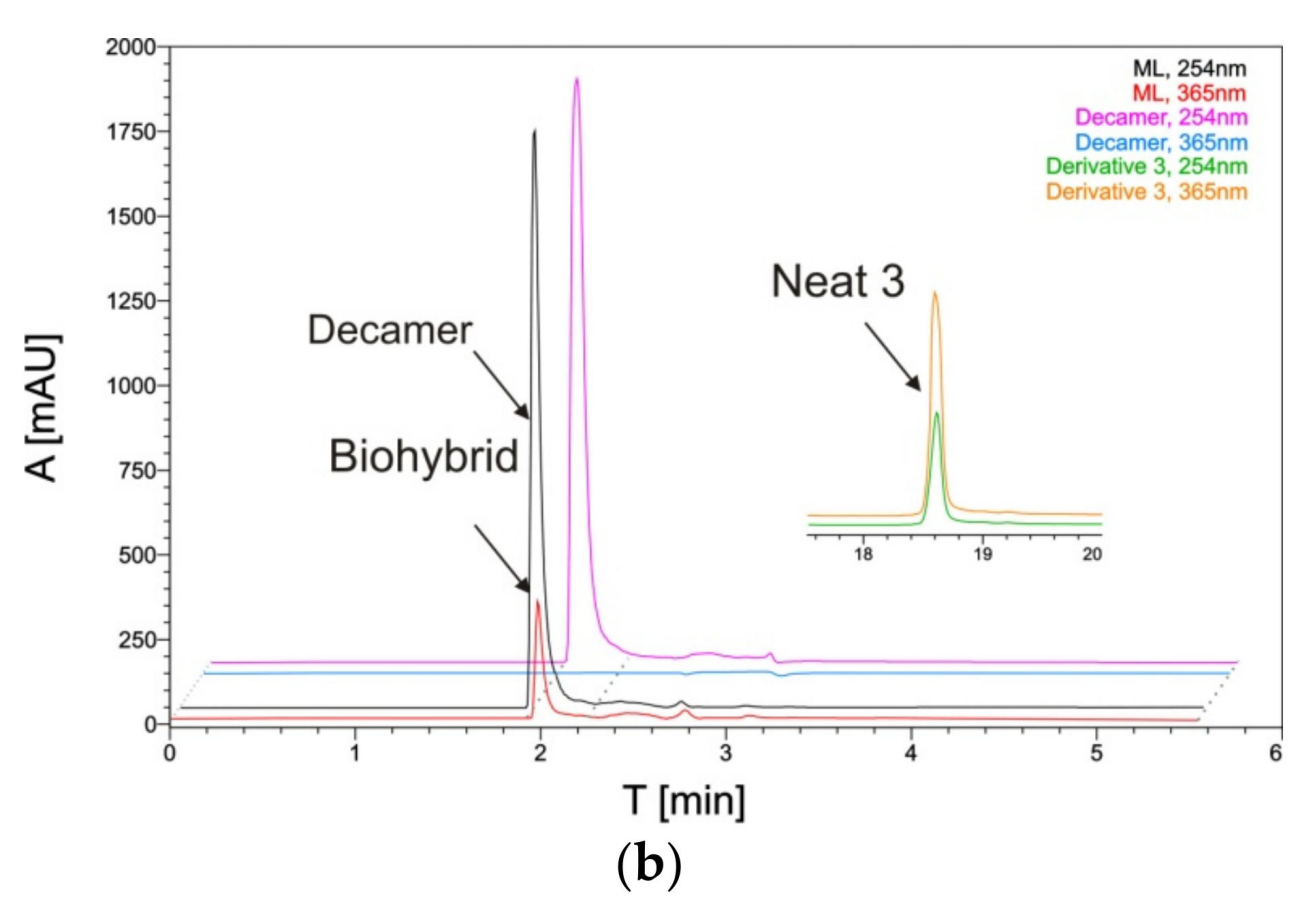
(**a**) The part of the DOSY spectrum of a 1:1 molar ratio of 1 and 3 in D2O buffer, pH 6, 25 °C in the mother liquor (ML) after filtration of the reaction mixture (Figure S6b). The diffusion coefficient Di, 1.0 ± 0.1 × 10^-10^ (m^2^ s^-1^) is equal for both components; (**b**) the part of the HPLC chromatogram run for the mother liquor as in DOSY, compared with neat decamer. The run monitored by 365 nm shows the same retention time of the compound bound to DNA. The inset shows the retention time for free compound **3**.

The same procedure of filtering was applied to the reaction mixture of compounds **1** and **3** (fully enriched 24-^13^C). The solid, which precipitated during reaction, was analyzed separately by HPLC (Figure S15). The DOSY and HPLC analysis results of the mother liquor (ML) for reaction with compound **3** are given in Figure 6 (see also Figure S15).

The DOSY experiment (Figure 6a) shows that compounds in the ML solution have the same diffusion coefficient, although the broadening of 19-CH_3_ signal may suggest that broadening is due to the overlap of this signal derived from a few compounds strongly bound to DNA and a covalently bound bioconjugate. It cannot be directly judged from this experiment which ligand is bound to nicked DNA, although the HPLC analysis suggests the presence of the bioconjugate (Figure 6b, peak with the retention time ca. 2 min), and bound compound 4 and SN38 (Figure S15), because any of these can persist when bound to **1** in solution.

**Figure 7:**
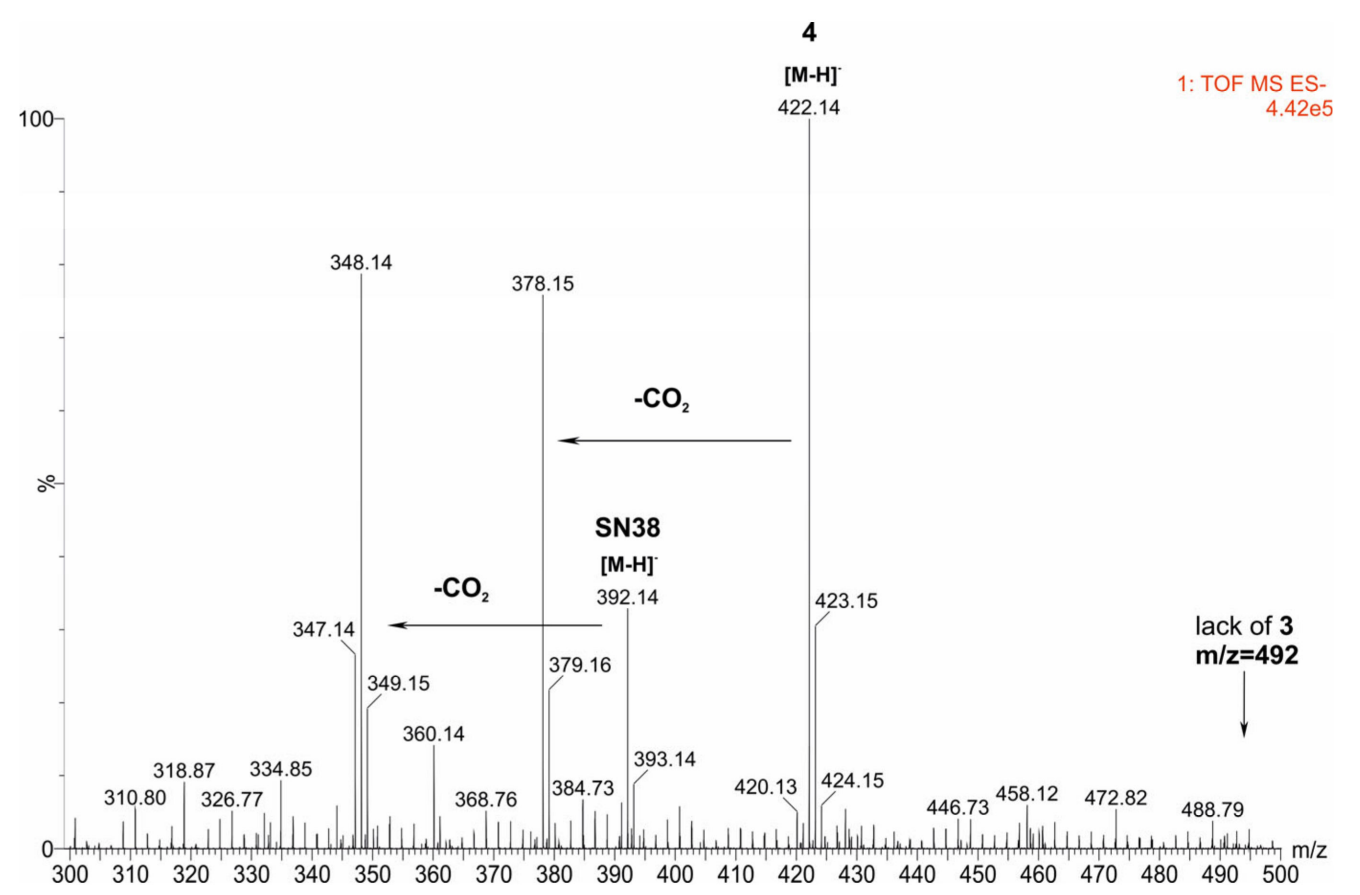
The ESI–MS spectrum (in the *m*/*z* range 300–500) of negative ions of a mother liquor of 24-^13^C-enriched compound 3.

The ESI–MS spectrum of ML in Figure 7 does not show the mother peak of **3** (*m*/*z* = 492) in positive and negative ions. The spectrum shows a dominant peak of compound **4** (*m*/*z* = 422) and SN38 (*m*/*z* = 391) as a product of the retro-Mannich reaction of **3** (Scheme 3). Both can be considered as ligands of a broken molecular complex with **1**. Consistent with this, the MALDI spectrum in the *m*/*z* range 5000–8000 (Figure S16a,b) shows a peak of neat DNA (*m*/*z* = 6888,2; [M + H]^+^) of a host molecule and peak (*m*/*z* = 7291.5, [M + H]^+^) of an alkylated biohybrid.

In view of the NOESY results and possible geometry established for the molecular complexes, which showed close contacts of the 24-C carbon atom of **2** with nucleophilic centers in the DNA nick, compound **3** was synthesized with fully ^13^C-enriched formalin (Figure S3a). This enabled the monitoring of the fate of the 24-C carbon atom during the reaction and provides preliminary information about the nitrogen base in the nick of **1** with the reactive methide intermediate of **3** as a site of alkylation [17]. Due to the low content of biohybrid **1** + **3** in the mother liquor, it was not possible to assign the site of alkylation and, therefore, we instead determined this by comparing the ^13^C chemical shift from the HSQC spectrum of the enriched 24-C^13^ in a biohybrid with the same carbon atoms in model nucleosides alkylated with **3**. 

**Figure 8:**
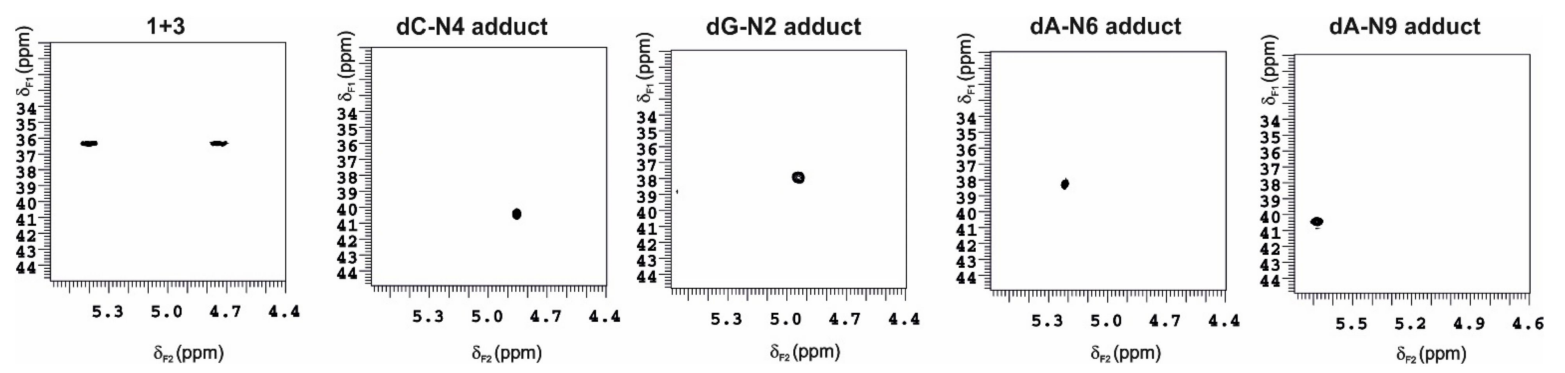
Partial ^1^H/^13^C HSQC spectra for biohybrid 1 + 3 of the present study and alkylated model 2′-deoxynucleosides.

Figure 8 presents a comparison of ^1^H/^13^C HSQC spectra of the relevant spectral ranges from the alkylation reaction of **1**, 2′-deoxyguanosine [18], 2′-deoxycytidine [19], and 2′-deoxyadenosine [20] with **3**. It can be seen that carbon chemical shifts of the 24-C atom fall in biohybrids within a narrow range of ca. 40 ppm.

The ^1^H chemical shift falls within the expected range of 4.6–5.5 ppm, although it can differ for reaction **1** + **3** from the model compounds because the former depends on various environmental effects in the nick, such as the anisotropy effect of aromatic rings or steric compression. These effects can also explain the observed nonequivalence of 24-CH_2_ protons. It can therefore be tentatively suggested that the observed HSQC cross-peaks reflect the alkylation of NH_2_, as in any of the model compounds. The molecular modeling discussion corroborates this tentative suggestion. It was shown (as is displayed in Figure 2 and Figure 3) that the 24-C carbon atom of intermediate *o*-methylene quinone of **2** is in close proximity to the NH_2_ protons in C15, A16, and G6.

In summary, the NMR experiments provide evidence of the interaction of alkylamino substituted in position 9-C SN38 derivatives, in the nick of the model nicked DNA duplex. The NMR, ESI–MS, and MALDI–MS experiments show that both compounds **2** and **3** react in a different way. It was shown that a strong molecular complex was formed with derivative **2** (*Ka* 4.07 mM^−1^) at the initial stage of the interaction, and it persists in a mother liquor (ML). In addition, a nucleophile in the nick is alkylated. Both of these phenomena confirm the strong binding of the target DNA, as confirmed by DOSY. The reaction products also contain the product of the hydrolysis reaction of derivative **2** and **3**. The ESI–MS of the ML does not show the presence of SN38 in the case of compound **2**. However, the ML of compound **3** shows the presence of the alkylated **1**, compound 4, and a minor amount of SN38. These coexisting phenomena are confirmed by DOSY, ^1^H/^13^C HSQC, and MALDI–MS experiments.

### 2.5. The Topo I Relaxation Activity Test

Human Topo I is a type I topoisomerase that is able to relax supercoiled DNA. In order to confirm the influence of studied compounds on topoisomerase I relaxation activity, we performed the test based upon the measurement of fluorescence of DNA stain which can differentiate between supercoiled and relaxed DNA.

**Figure 9:**
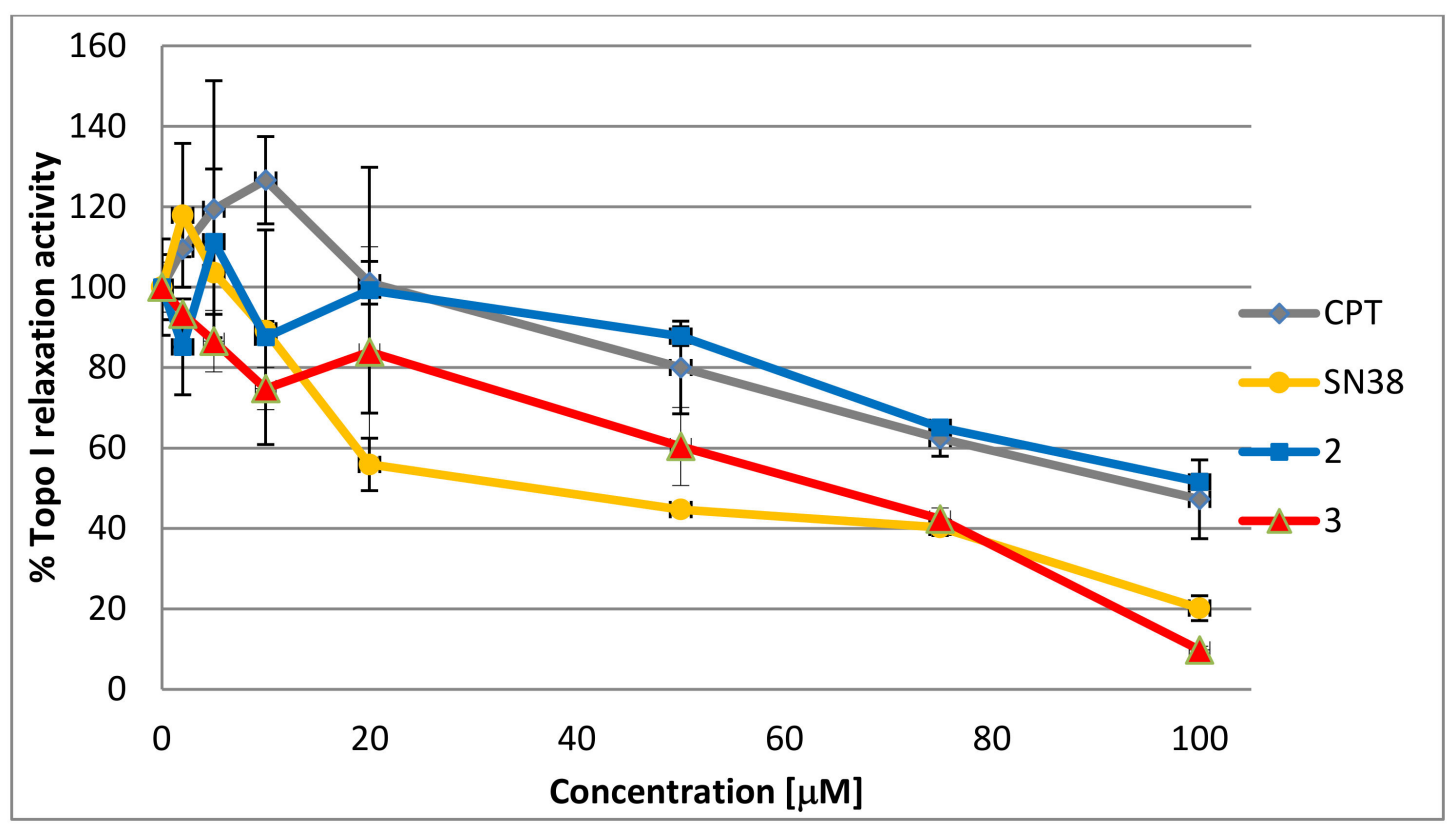
The inhibition of human Topo I relaxation activity with regard to increasing concentrations of campthotecin (CPT), SN-38 and new Topo I poisons 2 and 3. The mean and SD are presented on a graph.

Camptothecin (CPT) and SN38 are well-known Topo I interfacial inhibitors acting as Topo I poisons and were chosen as the positive control and reference standard. First, the amount of Topo I needed to just fully relax the supercoiled plasmid was determined. We then observed concentration-dependent change in the ratio of relaxed plasmid after the addition of camptothecin and SN38 to the reaction mixture, containing DNA plasmid and Topo I (Figure 9). The calculated IC_50_ indexes are equal to 92.9 and 40.3 µM (Table 6) for CPT and SN38, respectively. At the concentration of 100 µM, SN38 was able to reduce the Topo I relaxation activity by 80%, while camptothecin only by half.

Then, the relaxation activity of human Topo I was measured after the addition of SN38 derivatives. The obtained results shows that derivative **2** reduced Topo I relaxation activity as effectively as camptothecin. The IC_50_ index reached the value of 100.7 µM. The significantly better properties than camptothecin and compound **2** exhibits derivative **3**. The IC_50_ index calculated for **3** reached the value of 48.7 µM and was at the level established for SN38. Moreover, at the highest concentration (100 µM), the inhibition of Topo I relaxation activity was stronger for **3** than for SN38—the % of relaxed DNA plasmid was lower than 10% of initial amount, which is twice as much as that for SN38.

The presented results confirmed that studied SN38 derivatives effectively block DNA relaxation mediated by Topo I, which means that they stop the machinery of Topo I activity.

## 3. Materials and Methods

### 3.1. Chemical Substrates

The nicked decamer 1 was purchased from FutureSynthesis and purified by filtering on a membrane of 3 kDa. The compounds **2** (7-ethyl-9-(N-methylamino)methyl-10-hydroxycampthotecin) and **3** (7-ethyl-9-(N-morpholino)methyl-10-hydroxycampthotecin) were synthesized and purified as previously described [15]. The compounds enriched with ^13^C at the C-9 CH_2_ group were synthesized using the same prescription but using ^13^C-enriched formalin. 

### 3.2. Sample Preparation

Intermolecular interactions were studied in NMR sample tubes of solutions as described below.

Sample **1 + 2**: Decamer **1** (0.975 nmol dissolved in 650 µL in a buffer NaCl/K_3_PO_4_ 25 mM each in D_2_O or H_2_O/D_2_O; 1:9) was dissolved and 2.925 nmol of derivative **2** (7-ethyl-9-(N-methylamino)methyl-10-hydroxycamptothecin was added as a solid and pH adjusted to 6.

Sample **1 + 3**: Decamer **1** (0.900 nmol dissolved in 600 µL in a buffer NaCl/K_3_PO_4_ 25 mM each in D_2_O or H_2_O/D_2_O; 1:9) was dissolved and 1.800 nmol of derivative **3** (7-ethyl-9-(N-morpholino)methyl-10-hydroxycamptothecin was added as a solid and pH adjusted to 6.

### 3.3. HPLC Analysis

HPLC analysis was performed using an HPLC system from Shimadzu USA Manufacturing Inc. (Canby, OR, USA) consisting of a low-pressure gradient flow LC-20AT pump, a DGU-20A online solvent degasser, an SPD-M20A photodiode array detector, an SIL-10AF sample injector, and an FRC-10A fraction collector. Data were monitored using a Shimadzu LabSolution system. The analysis was carried out a Phenomenex Gemini 5 µm NX-C18 110 Å, 250 mm × 4.6 mm (or Phenomenex Gemini 5 µm NX-C18 110 Å, 250 mm × 10 mm) column, with the following mobile phases: (A) 10 mM NH_4_COOH aqueous solution, pH = 6; (B) MeCN with a gradient of 5–30% for 0–15 min, 30–50% for 15–20 min, and 50% after 20 min. The flow rate of the mobile phase: 1 mL/min (or 3 mL/min). The course of the chromatography was monitored using UV detection at a wavelengths of 260–365 nm. 

### 3.4. NMR Experiments

NMR spectra were recorded at 298 K on a Varian VNMRS-500 spectrometer equipped with a 5 mm Z-SPEC Nalorac IDG 500-5HT gradient probe. The experiments were performed in buffer solution (25 mM NaCl/25 mM K_3_PO_4_ in D_2_O). The spectra were referenced in D_2_O against TSPA-d_4_. Standard pulse sequences were used.

The experiments were performed under the following conditions:

TOCSY—spectral widths 5000 Hz in both dimensions, 750 complex points in t_2_, 512 complex points in t_1_, 64 scans per increment, 1 s relaxation delay, and 80 ms spin-lock time.

NOESY—spectral widths of 5000 Hz in both dimensions, 1024 complex points in t_2_, 512 complex points in t_1_, 64 scans per increment, 1 s relaxation delay, and 200 ms mixing time.

HSQC—spectral widths 8000 Hz in F2 and 22,600 Hz in F1, 1202 complex points in t_2_, 256 complex points in t_1_, 4 scans per increment, and 1 s relaxation delay.

Oneshot [29] DOSY spectra—1024 transients, 16 dummy scans, diffusion time (Δ) 150–220 ms, total diffusion encoding gradient duration (δ) 2 ms, and 16 values of the diffusion-encoding gradient incremented from 6 to 50 G/cm in such steps that the strength of the next gradient was equal to the previous gradient squared. Processing was carried out using the VARIAN VNMRJ software with the option of correction for spatially non-uniform pulsed field gradients.

### 3.5. ESI–MS Experiments

High-resolution ESI–MS spectra were acquired using an ultra-performance liquid chromatograph ACQUITY UPLC I-Class (Waters, Milford, MA, USA) coupled with a Synapt G2-S HDMS (Waters, Milford, MA, USA) mass spectrometer equipped with an electrospray ion source and q-TOF type mass analyzer. The instrument was controlled, and recorded data were processed using MassLynx V4.1 software package (Waters, Milford, MA, USA). The electrospray ionization–mass spectrometry (ESI–MS) spectra were recorded in the positive and negative ion mode in the *m*/*z* range 50–3000.

### 3.6. MALDI–MS Experiments

Mass spectra were acquired in negative or positive reflector mode on Applied Biosystems/MDS SCIEX MALDI 4800 Plus TOF/TOF spectrometer. Analyte ionization was achieved with a 355 nm Nd:YAG laser firing at 200 Hz rate. Laser fluence was within the 6000–6900 AU range. The analyzer was operated in delayed extraction mode from 250 to 650 ns. Typically, 1000 laser shots were accumulated. The raw spectrum was analyzed and edited (Gaussian smoothing, filter width: 99 points) using Data Explorer software, Applied Biosystems.

### 3.7. MALDI Sample Preparation

Solid sample (ca. 0.2 mg) was dissolved in water (ca. 20 μL) to reach the final concentration of about 10 mg/mL. A series of dilutions (with matrix solution) was then prepared: 1:1, 1:5, 1:10, 1:50 (*v*/*v*) ratio. As a matrix, a freshly prepared mixture of diammonium hydrogen citrate solution (50 mg/mL in water) and 3-hydroxypicolinic acid (50 mg/mL in acetonitrile/water 50:50 *v*/*v*) at a 1:8 *v/v* ratio was used. Aliquots (0.5 μL) of sample solutions were deposited on a stainless steel MALDI target plate and allowed to dry.

### 3.8. Calculating Binding Constants from the Diffusion Coefficients

The binding constants (K_a_) of the complexes were estimated by the analysis of the diffusion coefficient of DNA (1), ligand (compound **2**), and DNA–ligand complex as a function of the host and guest concentration [30] according to Equations (1) and (2):

(1) DNA+L ↔DNA·L

(2) $$K_{a}=\frac{\left[\mathrm{DNA}\cdot L\right]}{\left[\mathrm{DNA}\right]\left[L\right]}\;$$

In the case in which the exchange rate between the uncomplexed and complexed species was fast on the NMR timescale, the observed diffusion coefficients (D, (m^2^ s^−1^)) are a weighted average of the diffusion coefficients of the uncomplexed and complexed forms, where the weighting factors are the relative population sizes of the respective forms. Thus, the observed diffusion coefficients may be expressed as

(3) $$D_{\mathrm{OBS}-L}={\mathrm{MF}}_{L}D_{L}+\left(1-{\mathrm{MF}}_{L}\right)D_{\left[\mathrm{DNA}\cdot L\right]}$$

(4) $$D_{\mathrm{OBS}-\mathrm{DNA}}={\mathrm{MF}}_{\mathrm{DNA}}D_{\mathrm{DNA}}+\left(1-{\mathrm{MF}}_{\mathrm{DNA}}\right)D_{\left[\mathrm{DNA}\cdot L\right]}$$

where D_OBS-L_ and D_OBS-DNA_ are the observed averaged diffusion coefficients for L and DNA; D_L_ and D_DNA_ are the diffusion coefficients for uncomplexed L and uncomplexed DNA; MF_L_ and MF_DNA_ are the molar fractions of uncomplexed L and uncomplexed DNA in the solution containing both molecules; and D_[DNA·L]_ is the diffusion coefficient for the complex.

The K_a_ can be also expressed as

(5) $$K_{a}=\frac{\left[\mathrm{DNA}\cdot L\right]}{\left(C_{\mathrm{DNA}}-\left[\mathrm{DAN}\cdot L\right]\right)\left(C_{L}-\left[\mathrm{DNA}\cdot L\right]\right)}$$

where C_DNA_ and C_L_ are the initial concentrations of DNA and L.

The unknown complex concentration can be calculated from equations:

(6) $$\left[\mathrm{DNA}\cdot L\right]=\left(1-{\mathrm{MF}}_{\mathrm{DNA}}\right)C_{\mathrm{DNA}}\;$$

(7) $$\left[\mathrm{DNA}\cdot L\right]=\left(1-{\mathrm{MF}}_{L}\right)C_{L}$$

In the case in which the host molecule is much larger than the guest, it can be assumed that the diffusion coefficient of the host–guest complex is the same as that of the host molecule:

(8) $$D_{\left[DNA\cdot L\right]}≅D_{OBS-DNA}$$

By combining Equations (3), (5), (7), and (8), K_a_ can be determined.

### 3.9. Molecular Dynamics Calculations

The modified DNA decamer structure previously established by us [13] (PDB ID: **1G1N**) was used as the model structure for our DNA receptor. The SN38 derivative 2 compound was manually docked to the nick in this receptor in all four possible stacking orientations. Additionally, the two starting orientations of the bulky substituents of SN38 derivative **1** (9-CH_2_–NH–CH_3_ and 7-Et) relative to the remainder of SN38 derivative **2** compound were taken into consideration. This yielded a total of eight systems, which were then subjected to molecular dynamics (MD) calculations. Then, the trajectories—doubled due to the starting orientation of the substituents—were combined into one, which resulted in four distinct structures. All MD calculations were carried out using the AMBER 14 suite of programs [31]. The electrostatic potential (ESP) charges were obtained for both the SN38 derivative **2** compound and DNA linkers by the HF/6-31G* calculations using the Gaussian 09 program [32]. The RESP charges were then calculated by charge fitting with the multi-conformational procedure of the antechamber module implemented in Amber. The missing GAFF force field parameters were obtained using the parmchk module. Each complex was neutralized by adding Na^+^ cations, and then solvated by TIP3 water molecules with a spacing distance of about 12 Å around the system surface, creating a periodic box. All complexes were subjected to molecular dynamics (MD) simulations using the pmemd.cuda Amber 14 module with the NVIDIA GPU acceleration and mixed ff12SB-GAFF force field. The particle mesh Ewald (PME) method was used to treat long-range electrostatic interactions, and a 10 Å cutoff was applied to the nonbonded Lennard–Jones interactions. The SHAKE algorithm was applied to constrain all bonds involving hydrogen atoms and a 2 fs time step was used in the dynamics simulation. First, the systems were minimized in two stages: the first stage restrained the atomic positions of the solute and only relaxed the water, and the second stage released the restraint and allowed all atoms to relax (both with 10,000 minimization steps). The systems were then slowly heated to 300 K using an NVT ensemble and 1,000,000 steps with the Langevin dynamics temperature control (gamma_ln = 1.0). Then, the systems were carefully equilibrated at NPT ensemble simulations at 1 bar pressure with gamma_ln = 5.0. The equilibrations lasted until the system reached a converged density value, usually for 10–20 ns. Finally, the NPT production molecular dynamics were run for each of 8 trajectories for 500 ns of simulations. The trajectories with the only difference in orientations of the bulky substituents of SN38 derivative 2 were combined, and the four obtained trajectories corresponding to structures 1–4 were used for further calculations and analysis.

#### 3.9.1. Calculating Binding Free Energies (Enthalpies) Using MM-PBSA and MM-GBSA Methods

The combined MD trajectories were uniformly sampled, yielding 50,000 samples for each structure 1–4. The water and Na^+^ cations were stripped, and the binding free energies (enthalpies) were calculated using MM-PBSA and MM-GBSA methods [33] according to the following equations:

ΔG°_Bind,Solv_ = ΔG°_Bind,Vacuum_ + ΔG°_Solv,Complex_ − (ΔG°_Solv,Ligand_ + ΔG°_Solv,Receptor_)

Solvation free energies were calculated by either solving the linearized Poisson–Boltzmann or generalized Born equation for each of the three states (this provides the electrostatic contribution to the solvation free energy) and adding an empirical term for hydrophobic contributions:

ΔG°_Solv_ = ΔG°_electrostatic,ϵ = 80_ − ΔG°_electrostatic,ϵ = 1_ + ΔG°_hydrophobic_

ΔG°_Vacuum_ was obtained by calculating the average interaction energy between receptor and ligand, and taking the entropy change upon binding into account.

ΔG°_Vacuum_ = ΔE°_MM_ − TΔS°

where G°_Bind,Solv_ is the free energy of binding of solvated molecules; G°_Bind,Vacuum_ is the binding free energy in vacuum; G°_Solv,Complex_, G°_Solv,Ligand_, and G°_Solv,Receptor_ are the solvation free energy for complex, ligand, and receptor molecules; G°_Electrostatic_ is the electrostatic solvation free energy; G°_Hydrophobic_ is the hydrophobic (nonpolar) solvation free energy; E°_MM_ is the molecular mechanic energy; T is the temperature; and S° is the entropy.

The entropy contribution in our calculations was neglected because of the comparison of states of similar entropy. All free energy calculations were carried out using the mm_pbsa.pl script from AmberTools.

#### 3.9.2. PM7 Semi-Empirical Calculations

The combined MD trajectories were uniformly sampled to yield 5000 structures for each structure 1–4. The water and Na^+^ cations were stripped, the DNA was shortened to one GC, and one TA base pair complexed with a SN38 derivative on both sides, respectively. The remaining DNA base pairs were capped from the cut side with the phosphorane groups. The structures thus prepared were energy minimized with the PM7 method using the MOPAC2016 program [34]. The water solvent was approximated with the COSMO model [35].

#### 3.9.3. Cluster Analysis

The CPPTRAJ module implemented in the Amber package was used for cluster analysis. During cluster analysis, similar conformations were identified and grouped together. The cluster analyses were performed for structures used in PBSA/GBSA calculations and for structures from PM7 energy minimizations. During clustering analysis, the k-means clustering algorithm was used. The RMSD of heavy atoms was used as a distance metric calculated only for the SN38 derivative and the neighboring two DNA base pairs in each side for the structures from PBSA/GBSA calculations and one DNA base pair for structures from PM7 calculations. The clustering procedure was repeated several times, and each time the low-population strange structures were gradually removed. Finally, for each system, the most populated clusters were obtained. For each cluster, the average energies were calculated (PBSA/GBSA or PM7) and the most representative structures were determined.

### 3.10. The Topo I Relaxation Activity Assay

The inhibition of topoisomerase I relaxation activity was tested in triplicate with Human Topoisomerase I Relaxation High Throughput Plate Assay (Inspiralis Ltd., Norwich, UK) according to Vendor’s Protocol. Briefly, the test was based upon the measurement of fluorescence of DNA stain which can differentiate between supercoiled and relaxed DNA. The inhibition of human Topo I relaxation activity was measured in assay mixture containing Topo I and supercoiled DNA substrate (plasmid pNO1). At the first step—the activity of Topo I—the amount of enzyme needed to fully relax the plasmid was determined. Then, for this specific amount of enzyme. the relaxation inhibition potency of the studied compound was tested. The increasing amounts of compounds (2–100 µM) were added to the reaction mixture together with the positive control and reference standard: camptothecin and SN38, respectively. The fluorescence of DNA plasmid stained with Diamond™ Nucleic Acid Dye (Promega, Madison, WI, USA) was read in fluorescence plate reader (495 nm/537 nm) [36,37].

## 4. Conclusions

It can be concluded that both parent compounds **2** and **3** yield some alkylated DNA. Compound **2** forms mainly a very stable and strong molecular complex with **1**, whereas compound **3** more readily undergoes hydrolysis and retro-Mannich reaction to yield compound **4** and SN38 (Scheme 3). It is worth mentioning that both phenomena, alkylation and strong intercalation inside the nick, can play an important, cooperative role in Topo I inactivation. The alkylation of bases in a nick eliminates the tumor DNA from further proliferation because it poisons the role of Topo I, i.e., the relegation of a broken strand in a duplex DNA by trapping a cleavable complex. In addition, the strong molecular complex of 2–4 and SN38, simultaneously and in a complementary fashion, can prolong the lifetime of a ternary complex Topo I/nicked DNA/inhibitor and prevent the restoration of the double-stranded unstrained DNA. The most important information resulting from this discussion concerning the safety of eventual chemotherapy using the studied compounds as potential medicines in vivo is the lack of SN38 in the interaction of **2** with the nicked DNA as a model of the biological target of Topo I poisons. It is well recognized that the toxicity of SN38—as the main metabolite of Irinotecan, which is currently used in clinics—is a cause of lethal diarrhea [38].

The molecular modeling based on cross-peaks in the NOESY spectrum allows the geometry of molecular complex **1** + **2** to be assigned. The results indicate the two possible complexes inside the nick (structures 1 and 3). Both face the unbroken strand with ring A and are equally included inside the interior of the nick. These results provide plausible support to the suggestions derived from the experiments. The geometry of structure 1 is also comparable with the X-ray structure of the ternary complex Topo I/DNA/TPT. Considering the promising results concerning the role of the studied compounds in interaction with the biological target of Topo I interfacial inhibitors, the studied compounds can be considered to be potential Topo I poisons for safe targeted chemotherapy, and further research for the development of both compounds is planned.

In order to confirm the influence of studied compounds on the topoisomerase I relaxation activity of supercoiled DNA, we performed the test based upon the measurement of fluorescence of DNA stain which can differentiate between supercoiled and relaxed DNA. The presented results confirmed that studied SN38 derivatives effectively block DNA relaxation mediated by Topo I, which means that they stop the machinery of Topo I activity.

## Figures and Tables

**Table 1:** The diffusion constants Di × 10^−10^ (m^2^ s^−1^) and binding constant calculation of a complex **1** + **2** from the DOSY data.

Sample	Concentration (mM)		1 (T7-CH_3_) Integral	2 (19-CH_3_) Integral	D*_i_* of 1 (10^−10^ m^2^ s^−1^)	D*_i_* of 2 (10^−10^ m^2^ s^−−1^)	K_a_ (mM^−1^)	% of Complex
	1	2						
Complex of 1 + 2 after 5 days	1.5 ^a^	3.25 ^b^	3	6.5	1.147	1.475	4.07	41% ^c^ 89% ^d^
Compound 2	-	2.0	-	-	-	3.07	-	-
Decamer 1	0.96	-	-	-	1.13	-	-	-

^a^ Concentration of **1** calculated from weighted amount (see experimental); ^b^ concentration of **2** calculated from the integral of 19-CH_3_ and T7-CH_3_ signals; ^c^ counted based on concentration of **2**; and ^d^ counted based on concentration of **1**.

**Table 2:** The ^1^H NMR chemical shift changes in SN38 derivative **2** after addition to DNA.

Compound 2	Free; δ (ppm)	Complex 1 + 2; δ (ppm)	Δ (ppm)	Δ (Hz)
19-CH_3_	0.96	0.993	0.033	16.5
23-CH_3_	1.50	1.377	−0.123	−61.5
18-CH_2_	1.95	1.908	−0.042	−21
N–CH_3_	2.82	2.894	0.074	37
22-CH_2_	3.09	2.911	−0.179	−89.5
24-CH_2_	4.54	4.512	−0.028	−14
24-CH_2_	4.62	4.512	−0.108	−54
5-CH_2_	5.02	4.65	−0.370	−185
17-CH_2_	5.36	5.259	−0.101	−50.5
17-CH_2_	5.51	5.259	−0.251	−125.5
14-CH	7.18	7.041	−0.139	−69.5
11-CH	7.32	7.081	−0.239	−119.5
12-CH	7.71	7.333	−0.377	−188.5

**Table 3:** The ^1^H NMR chemical shifts δ (ppm) of changes between free DNA decamer and **2** after 4 days in D_2_O (1:2 ratio). * (Table S2).

Base	H1’	H2’	H2”	H3’	H4’	H5’, H5”	H6/8	H2/5/Me
T4	−0.008	−0.026	−0.048	0.014	−0.035	-/−0.003	−0.005	−0.023
T5	0.020	**0.052**	−0.046	−0.006	−0.009	−0.001/-	**0.067**	−0.012
**nick**	-----------	----------	----------	-----------	----------	------------------	-----------	-----------
G6	**−0.342**	**−0.284**	**−0.068**	0.017	−0.037	**−0.076/−0.222**	0.020	-
T7	−0.045	−0.022	−0.037	−0.018	**−0.082**	-/0.013	**−0.204**	−0.025
A14	−0.044	−0.035	**−0.128**	**−0.118**	**−0.057**	-/-	**−0.093**	**−0.058**
C15	**0.170**	0.010	**−0.199**	-	0.021	-/0.000	0.027	**−0.245**
A16	**−0.230**	0.024	**−0.093**	−0.043	0.029	-/-	**−0.224**	**−0.204**
A17	−0.040	−0.048	−0.031	−0.026	−0.015	**-/0.073**	**−0.069**	**−0.081**

* Bold is used to highlight the largest changes observed and red to highlight the protons involved in intermolecular cross-peaks.

**Table 4:** The analysis of measured distance statistics for selected protons in conformers recorded during MD trajectories for four possible structures with observed intermolecular NOE effects.

Deriv. 2	DNA 1	NOE ^(a)^	Structure 1 ^(b)^				Structure 2 ^(b)^				Structure 3 ^(b)^				Structure 4 ^(b)^			
			<6Å	<5Å	<4Å	<3Å	<6Å	<5Å	<4Å	<3Å	<6Å	<5Å	<4Å	<3Å	<6Å	<5Å	<4Å	<3Å
11	C15H2”	8.0%	++++	++++	++++	++					++++	+++	+	*				
12	A16H8	8.4%	++++	++++	+++						+++	+						
14	A16H2	23.5%	++++	++++	++++	+++	++								++++	++++	++++	+
17a	G6H1′	11.0%	++++	++++	++++	+++					+							
17b			++++	++++	+++	.					++++	++	*					
17a	G6H4′	16.2%	++++	++++	++++	+++					++	.						
17b			++++	++++	++++	+					++++	++	++	*				
17a	T5H1′	6.9%	++++	+++	*						+	*						
17b			++++	++++	+++	+					+							
17a	T5H2”	14.8%	++++	++++	+++						+++	++	+	*				
17b			++++	++++	++++	+++					++++	+++	+					
17a	T5H2′	9.4%	++++	+++	+						++++	+++	++	*				
17b			++++	++++	++++	++					++++	++++	+					
17a	T5H6	4.7%	++++	+							++++	++++	++					
17b			++++	++++	+++						++++	++++	*					
18	G6H1’	12.3%	++++	++++	+++	++	*								+	*		
18	G6H2”	9.1%	+++	++	*													
18	G6H4′	11.7%	++++	+++	++	+									*			
18	T7H4′	5.7%	++++	+++	++	*									*	*		
18	T7H5”	4.7%	++++	+++	+										*			
18	T7H5′	11.5%	++++	++++	+++	++									*	*		
19	A16H2	2.3%	+++	*											++++	++++	++	*
19	G6H1′	5.4%	++++	+++	++	*									*			
19	G6H2”	4.1%	+++	++	*													
19	G6H4′	7.9%	++++	+++	++	*									*			
19	T7H4′	12.1%	++++	++++	++++	+									*	*		
19	T7H5”	5.7%	++++	+++	++										*	*		
19	T7H5′	10.9%	++++	++++	+++	++									*	*		
24	C15H5	5.7%	++++	++++	++	+												
N–CH_3_	C15H6	1.7%	++	*							*							

^(a)^ Percentage of the intermolecular NOE effects measured by integrating the relative cross-peaks. between given protons compared to NOE between protons 11 and 12 of SN38 derivative **2**. ^(b)^ ++++, +++, ++, +, and * indicate, respectively: >50%, >25%, >10%, >5%, and >1% (more than) MD sampled conformers for a given structure that meets the corresponding distance condition between indicated protons.

**Table 5:** The interatomic distances between atoms potentially interacting to form a hybrid (Figures S11 and S12).

**Atom Names**		**Atom Distances in Structure 1 in (Å)**		
**DNA**	**Comp. 2**	**100% Cluster ^a^**	**50% Cluster ^a^**	**25% Cluster ^a^**
C15-N4	-CH_2_NHCH_3_	7.23 ± 12.64	3.90 ± 0.33	3.62 ± 0.21
A16-N7	-CH_2_NHCH_3_	7.53 ± 12.78	4.27 ± 0.32	4.01 ± 0.25
A16-N6	-CH_2_NHCH_3_	8.47 ± 12.79	5.12 ± 0.49	4.75 ± 0.44
		**Atom Distances in Structure 3 in (Å)**		
G6-N2	-CH_2_NHCH_3_	6.43 ± 10.88	4.03 ± 0.32	3.76 ± 0.21
A16-N3	-CH_2_NHCH_3_	5.51 ± 10.65	3.37 ± 0.13	3.26 ± 0.09

^a^ Represents the population of a cluster encompassing structures with shortest relevant distances between interacting atoms to form hybrid.

**Table 6:** The IC_50_ indices—the concentration required to reduce the relaxation Topo I activity by 50%.

IC_50_ (µM)			
CPT	SN38	**2**	**3**
92.9 ± 16.1	40.3 ± 13.17	100.7 ± 10.0	48.7 ± 8.2

## Data Availability

The authors confirm that the data supporting the findings of this study are available within the article and its Supplementary Materials.

## Supplementary Materials

The following are available online at https://www.mdpi.com/article/10.3390/ijms22147471/s1. Figure S1: The ^1^H NMR spectrum of nicked decamer 1; Figure S2: The ^1^H NMR spectrum of 2; Figure S3a,b: The ^1^H NMR spectrum of 3 and 4; Figures S4 and S5: DOSY spectrum of compounds 1 and 2; Figure S6a,b: DOSY spectrum of compounds 1 and 3; Figure S7: NMR spectrum of reaction mixture of 2 with 1; Table S1: The ^1^H NMR chemical shifts of DNA 1 with 2; Table S2: Chemical shifts changes of DNA decamer; Figures S8 and 8a: NOESY spectrum and cross-peaks in complex 1 + 2; Figure S9: NOESY spectrum of the sample 1 + 3; Figure S10: The example of the cross-peaks in sample 1 + 3; Figures S11 and S12: The best structure from modeling showing potential sites of hybrid formation in a molecular complex 1 + 2; Table S3: The most populated cluster energy from PBSA and GBSA analysis; Table S4: The real hydrogen bonds in 1 + 2 complex from PM7 calculations; Figure S13: The hydrogen bonding in structures best representing the most populated cluster in a complex 1 + 2; Figure S14: The MALDI–MS spectrum of ML 1 + 2; Figure S15: HPLC chromatograms for reaction 1 + 3; Figure S16a,b: The MALDI–MS spectrum of the ML from reaction 1 + 3.
