# Supplementary material for: The Mode of SN38 Derivatives Interacting with Nicked DNA Mimics Biological Targeting of Topo I Poisons
Source: Int J Mol Sci. 2021 Jul 12;22(14):7471. doi: 10.3390/ijms22147471 (PMC8303725; doi:10.3390/ijms22147471)
Supplement: Supplementary file 1 [file ijms-22-07471-s001.zip › ijms-1293299-supplementary.pdf]

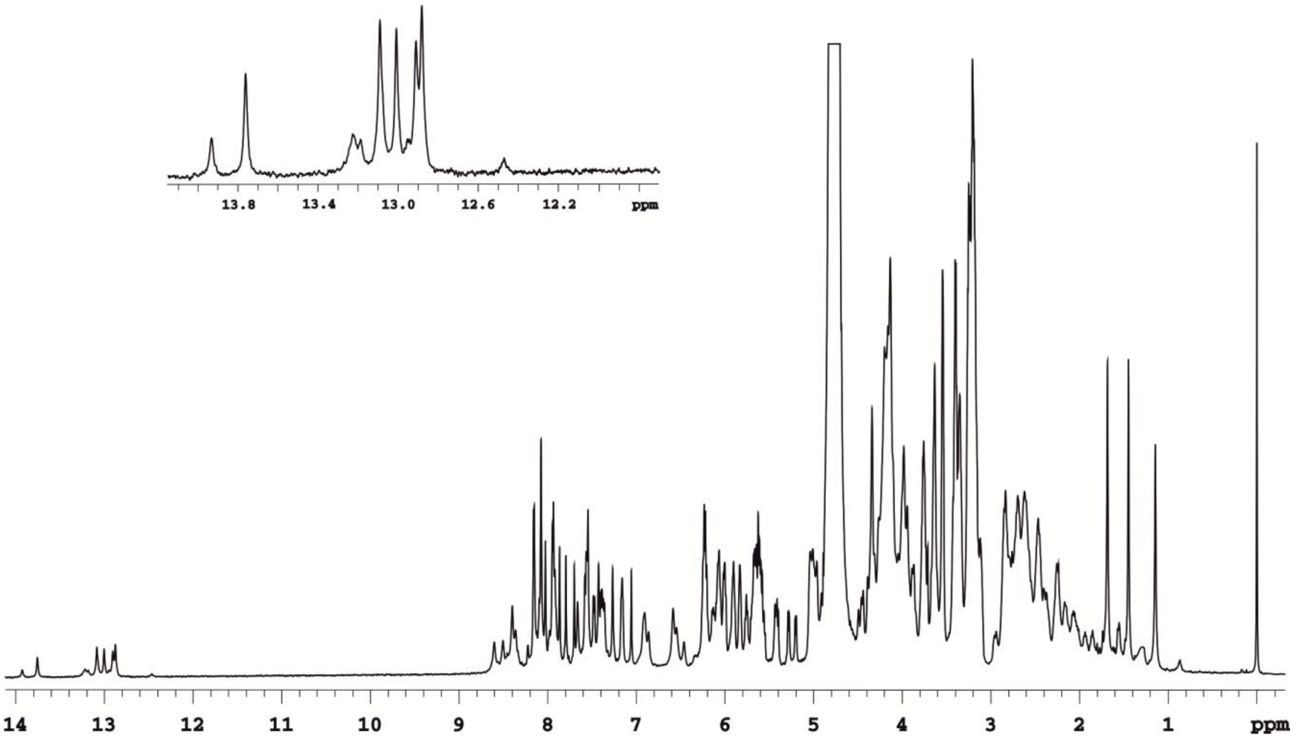

Fig. S1 The ^{1}H NMR spectrum of nicked decamer **1** in buffered H_2O/D_2O, (90/10 vol%) 25 mM NaCl /25 mM K_3PO_4, at pH 6. Seven guanosine and 3 tymidine NHs hydrogen bonded, forming a duplex are shown in an inset.

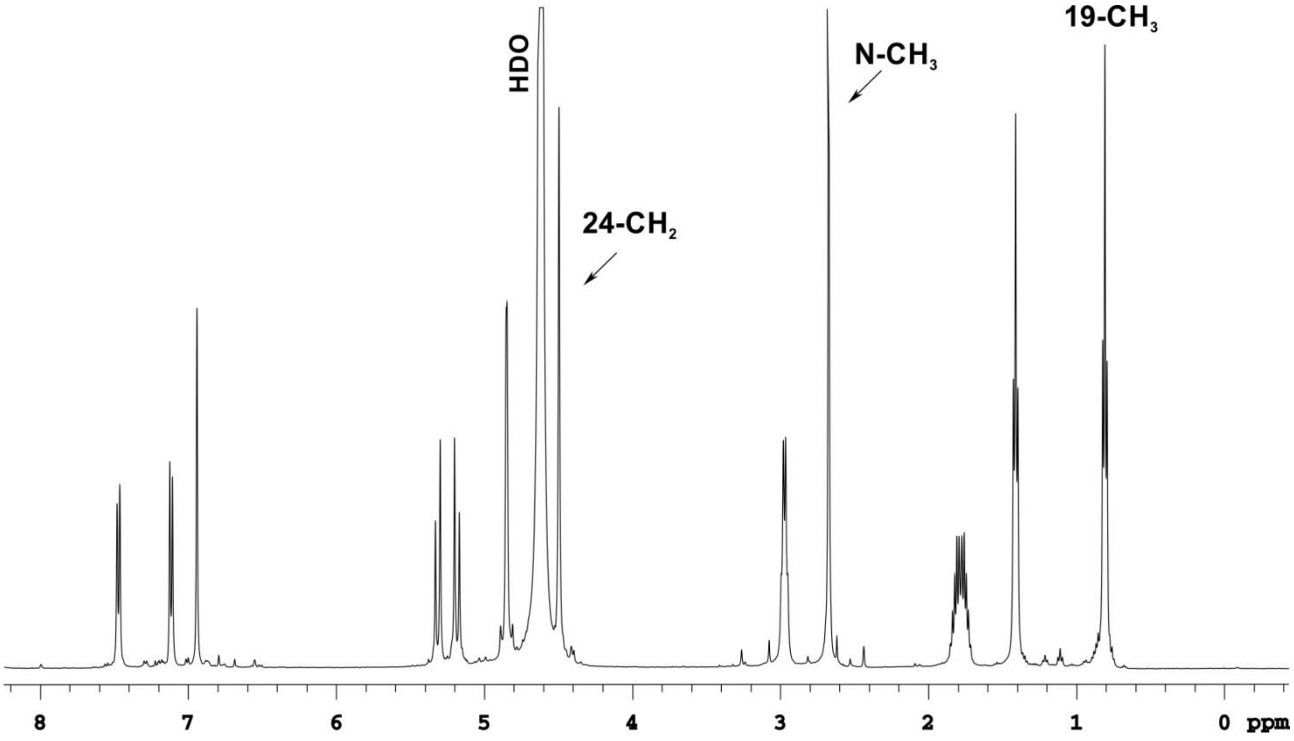

Fig. S2 The ^{1}H NMR spectrum of **2** in buffered $/D_2O$, 25 mM NaCl /25 mM K_3PO_4, at pH 6.

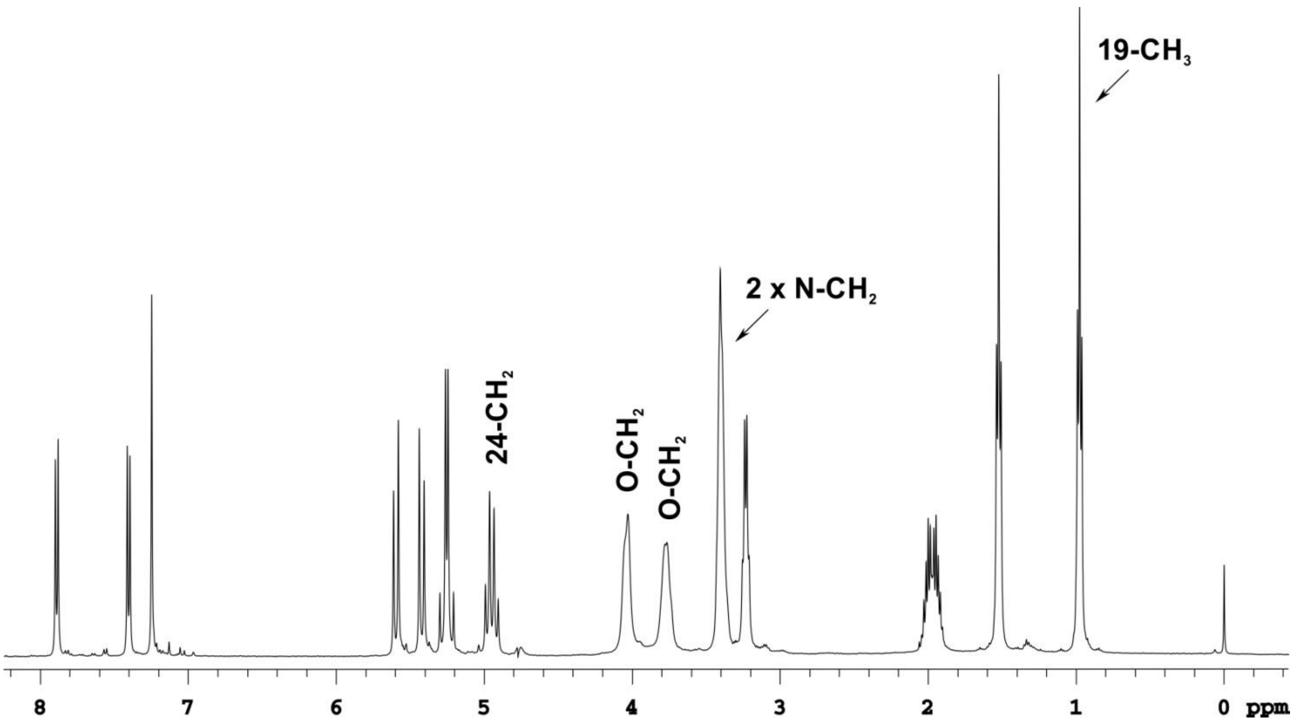

Fig.S3 The ^{1}H NMR spectrum of **3** in buffered D_2O, 25 mM NaCl /25 mM K_3PO_4, at pH 6.

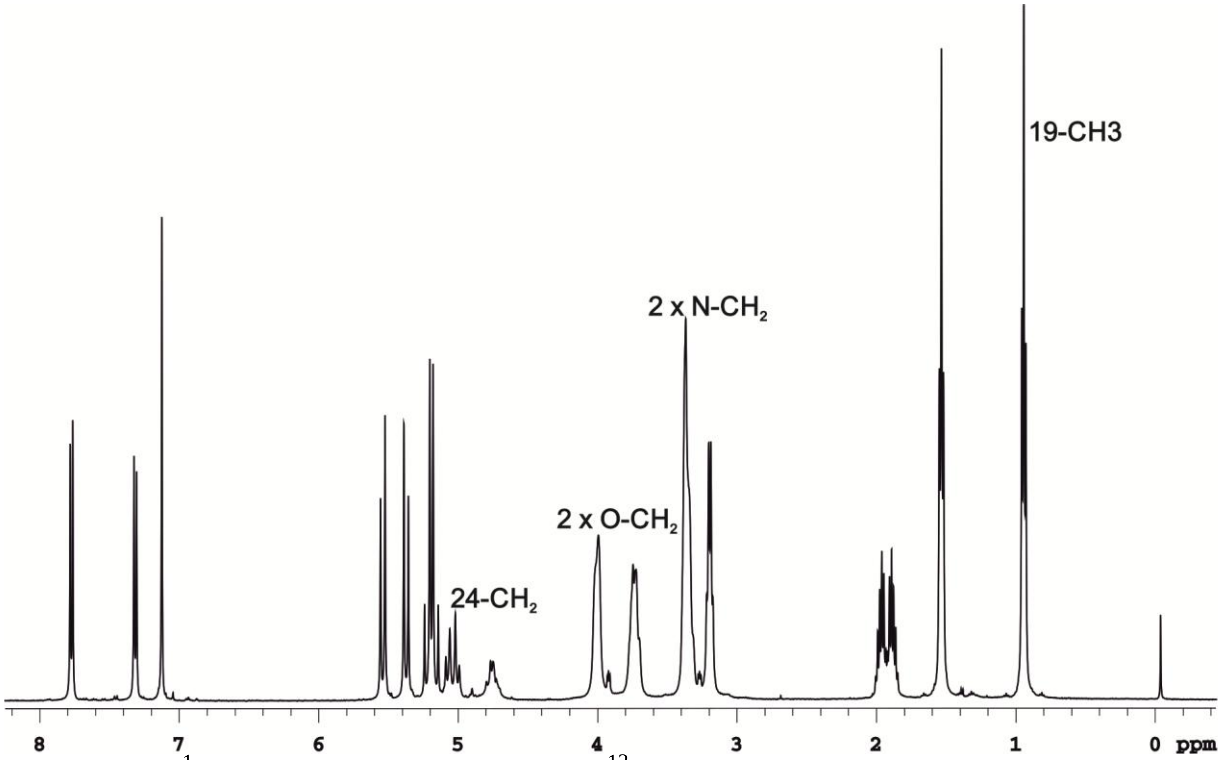

Fig.S3a The ^{1}H NMR spectrum of **3** enriched ^{13}C-24 in buffered D_2O, 25 mM NaCl /25 mM K_3PO_4, at pH 6.

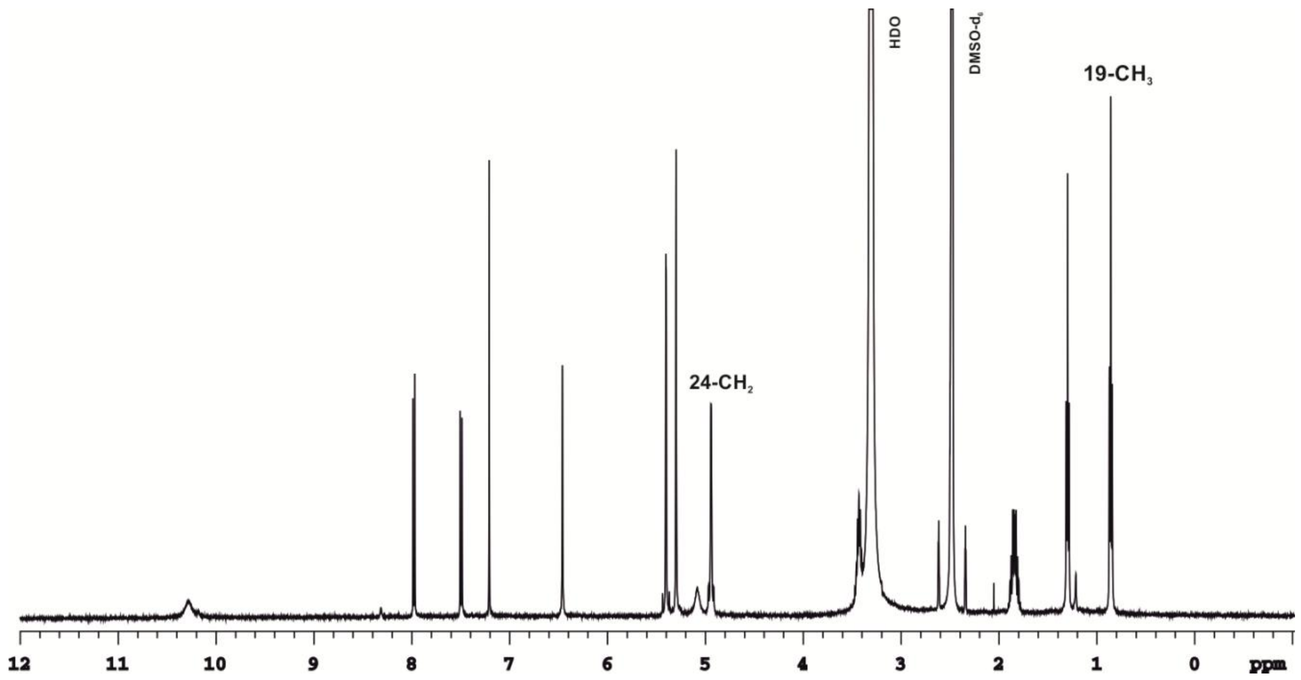

Fig. S3b The ^{1}H NMR spectrum of metabolite **4** in DMSO-d_6.

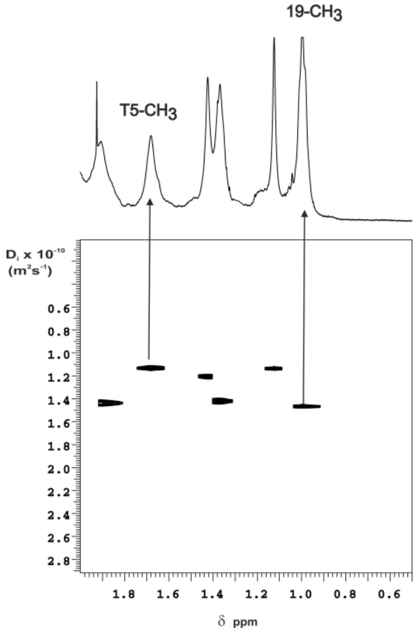

Fig. S4 The part of the DOSY spectrum presenting result for compounds **1** and **2** after 5 days of incubation in buffered D_2O, 25 mM NaCl /25 mM K_3PO_4, at pH 6.

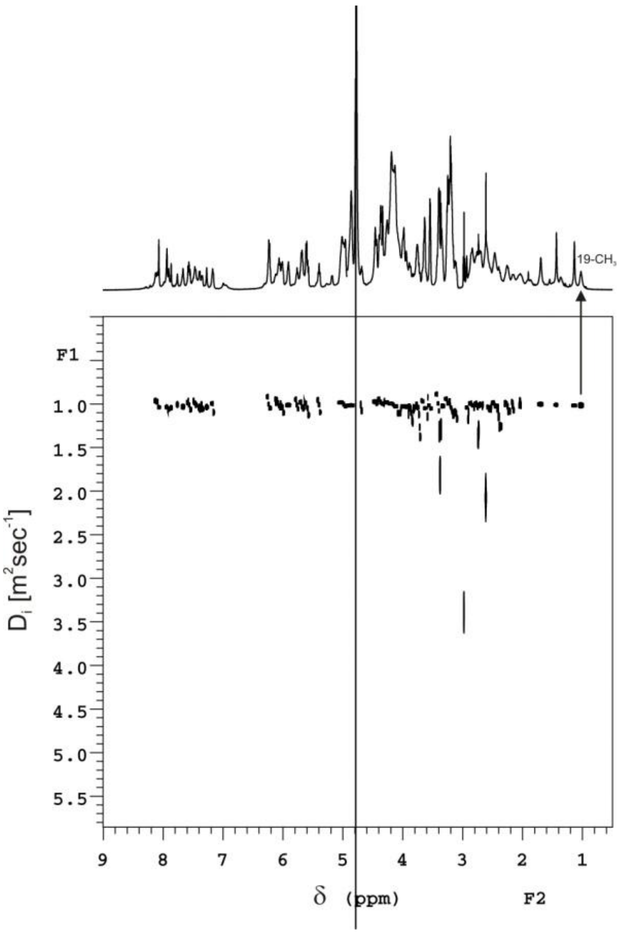

Fig. S5 The full DOSY spectrum for decamer **1** and derivative **2** after filtering the sample of reaction solution, ML . The DOSY spectrum of a 1:1 complex of **1** and **2** in D_2O buffer, pH 6, 25^oC. The diffusion coefficient D_i, 1.0 ± 0.1 x 10^{-10} [m^2s^{-1}] is equal for both components.

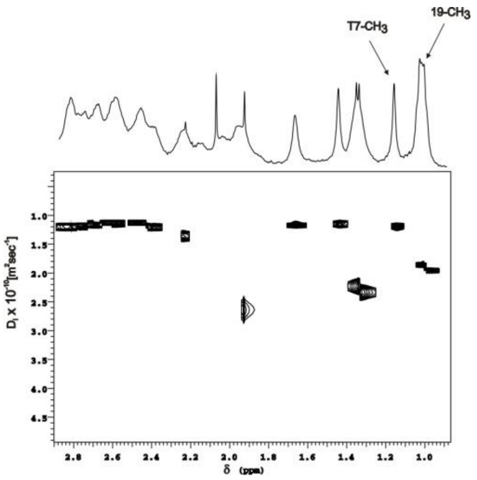

Fig. S6a The part of the DOSY result of compounds **1** and **3** at the start, in buffered D_2O, 25 mM NaCl /25 mM K_3PO_4, at pH 6.

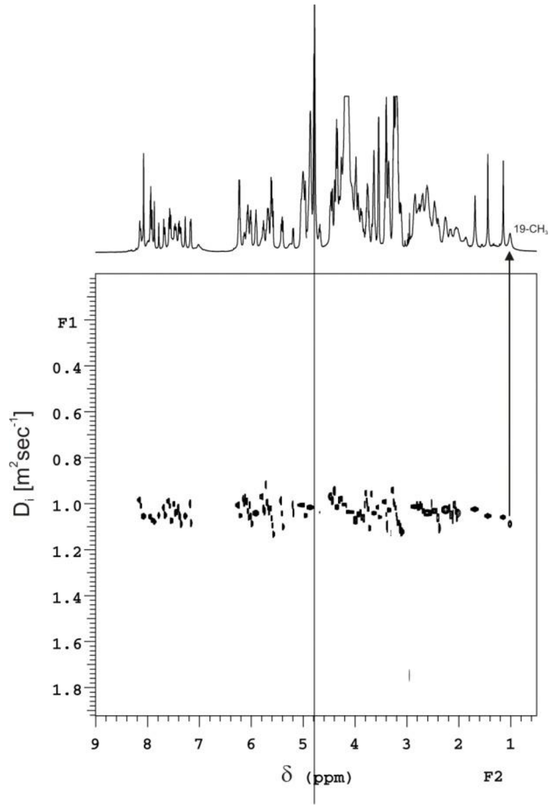

Fig.S6b The full DOSY spectrum for decamer 1 and derivative **3** after filtering the sample of reaction solution.

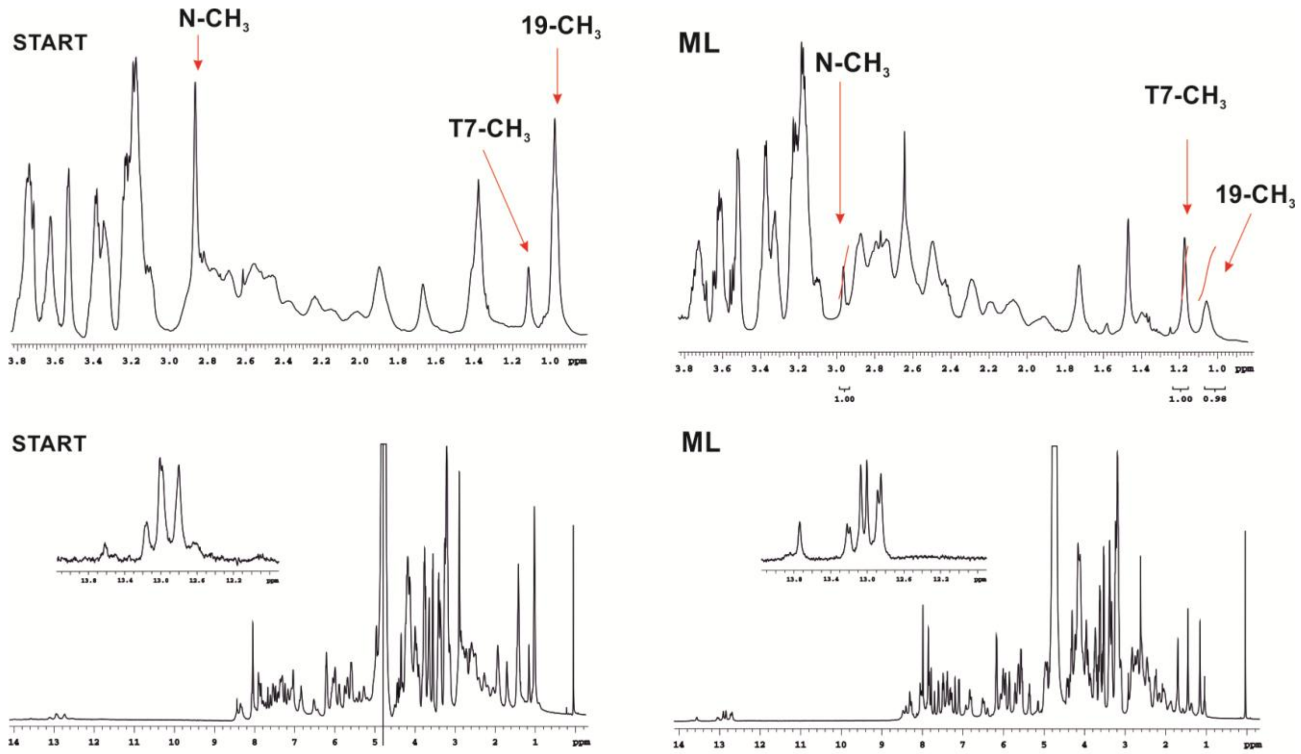

Fig. S7 The shape of 1D NMR spectrum of reaction mixture of **2** with **1** at the start and in a mother liquor ML after filtering of reaction solution. Expansion of low frequency region of a spectrum is given in both cases.

Table S1. The ^{1}H NMR chemical shifts δ [ppm] of DNA **1** and SN38 derivative **2** from NOESY spectrum in buffered D_2O, at 25°C **(1:2** ratio) acquired after 4 days of incubation the sample at 25 ^{o}C. *

Base	H1’	H2’	H2”	H3’	H4’	H5’, H5”	H6/8	H2/5/Me
G1	5.998	2.760	2.833	4.955	4.334	3.883/ 3.991	8.065	-
C2	5.692	2.140	2.439	4.860	4.217	4.161	7.384	5.380
G3	6.059	2.680	2.820	5.008	4.430	4.112/ 4.156	7.923	-
T4	6.055	2.058	2.559	4.885	4.239	na/ 4.310	7.263	1.423
**T5**	**6.222**	**2.550**	**2.571**	**4.984**	**4.191**	**4.098/ na**	**7.492**	**1.679**
nick	-----------	-----------	-----------	-----------	-----------	-----------------------	-----------	-----------
**G6**	**5.490**	**2.358**	**2.574**	**4.687**	**4.170**	**3.798/ 3.911**	**7.685**	**-**
T7	6.023	2.158	2.520	4.853	4.235	4.003/ 4.114	7.338	1.122
C8	5.742	2.021	2.391	4.853	4.111	na/ na	7.439	5.590
G9	5.898	2.626	2.695	4.999	4.364	4.075/ 4.133	7.925	-
C10	6.225	2.239	2.466	4.851	4.192	4.139/ 4.249	7.565	5.598

Base	H1’	H2’	H2”	H3’	H4’	H5’, H5”	H6/8	H2/5/Me
G11	6.002	2.755	2.828	4.955	4.333	3.880/ 3.991	8.064	-
C12	5.679	2.002	2.376	4.850	4.172	4.168	7.338	5.384
G13	5.673	2.757	2.778	5.032	4.391	4.078/ 4.156	7.894	-
A14	6.203	2.646	2.820	4.913	4.436	na/ na	8.065	7.739
**C15**	**5.733**	**1.948**	**2.163**	**na**	**4.165**	**na/ 4.303**	**7.183**	**5.045**
**|**								
**A16**	**5.601**	**2.598**	**2.715**	**4.965**	**4.387**	**na / na**	**7.806**	**6.854**
A17	6.093	2.591	2.807	5.011	4.444	na/ 4.289	8.092	7.622
C18	5.584	1.856	2.272	na	4.124	na/ 4.240	7.168	5.176
G19	5.889	2.583	2.686	4.972	4.337	4.038/ 4.118	7.858	-
C20	6.213	2.232	2.458	4.849	4.190	4.127 / 4.235	7.550	5.576

* The G6-C15 and T5-A16 base pairs, in bold, are flanking both faces of a nick.
na-not assigned.

Table S2. The ^{1}H NMR chemical shifts δ [ppm] of changes induced in free DNA decamer and **2** after 4 days incubation in D_2O (**1**:**2** ratio).

Base	H1'	H2'	H2"	H3'	H4'	H5', H5"	H6/8	H2/5/Me
G1	-0.006	-0.013	-0.006	-0.004	-0.009	0.001 / -0.020	-0.013	-
C2	-0.003	-0.011	-0.020	-0.008	-0.010	0.001	-0.007	-0.027
G3	-0.029	-0.021	-0.034	-0.013	-0.008	-0.004 / -0.009	-0.024	-
T4	-0.008	-0.026	-0.048	0.014	-0.035	-/ -0.003	-0.005	-0.023
T5	0.020	**0.052**	-0.046	-0.006	-0.009	-0.001 / -	**0.067**	-0.012
nick	-----------	-----------	-----------	-----------	-----------	----------------------	-----------	-----------
G6	**-0.342**	**-0.284**	**-0.068**	0.017	-0.037	**-0.076 / -0.222**	0.020	-
T7	-0.045	-0.022	-0.037	-0.018	**-0.082**	-/ 0.013	**-0.204**	-0.025
C8	-0.018	-0.045	-0.027	-0.015	-0.022	- / -	-0.036	-0.046
G9	-0.006	0.012	0.003	0.009	0.010	-0.003 / -0.001	-0.013	-
C10	-0.002	-0.017	0.005	0.005	-0.009	0.001 / -0.003	-0.007	-0.018

Base	H1'	H2'	H2"	H3'	H4'	H5', H5"	H6/8	H2/5/Me
G11	-0.002	-0.018	-0.011	-0.004	-0.010	-0.002 / -0.020	-0.014	-
C12	0.016	-0.013	-0.009	-0.001	-0.009	0.024	-0.029	-0.044
G13	0.012	0.019	-0.037	-0.015	-0.003	0.014 / 0.010	-0.016	-
A14	-0.044	-0.035	**-0.128**	**-0.118**	**-0.057**	- / -	**-0.093**	**-0.058**
C15	**0.170**	0.010	**-0.199**	?	0.021	- / 0.000	0.027	**-0.245**
A16	**-0.230**	0.024	**-0.093**	-0.043	0.029	- / -	**-0.224**	**-0.204**
A17	-0.040	-0.048	-0.031	-0.026	-0.015	-/ **0.073**	**-0.069**	**-0.081**
C18	-0.018	0.001	-0.007	?	-0.018	-/ -0.017	0.001	-0.024
G19	-0.015	-0.031	-0.006	-0.018	-0.017	0.008 / -0.001	-0.010	-
C20	-0.014	-0.024	-0.003	0.003	-0.011	-0.003 / -0.005	-0.005	-0.014

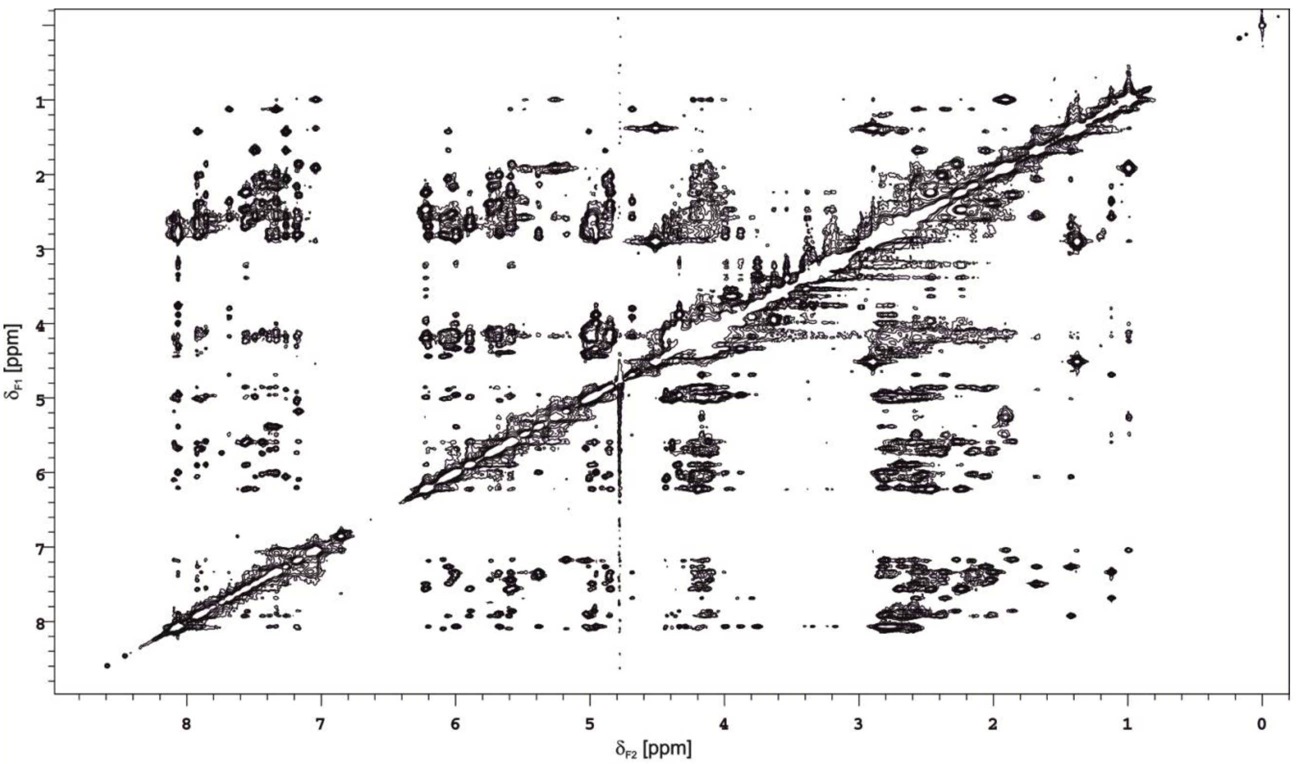

Fig. S8 The NOESY spectrum of the sample 1+2 after 4 days of incubation (**1**:**2** ratio).

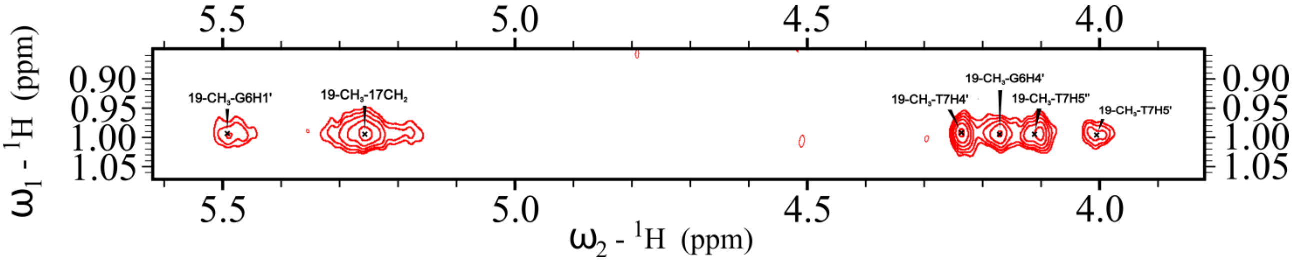

Fig. S8a The example of intermolecular cross-peaks in complex 1+2 after 4 days of incubation(**1:2** ratio), before filtering the reaction solution ,see Table 1 in manuscript.

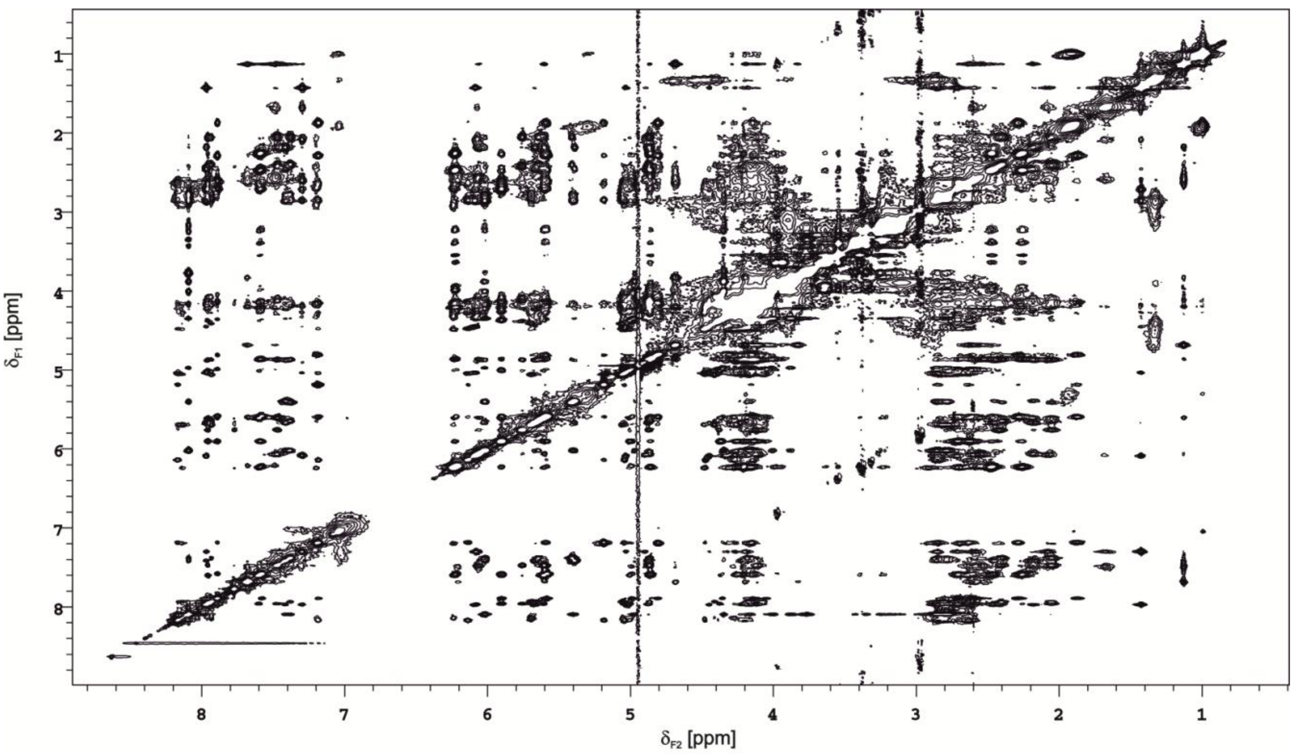

Fig S9 The NOESY spectrum of the sample 1+3 (see experimental section) after 24h.

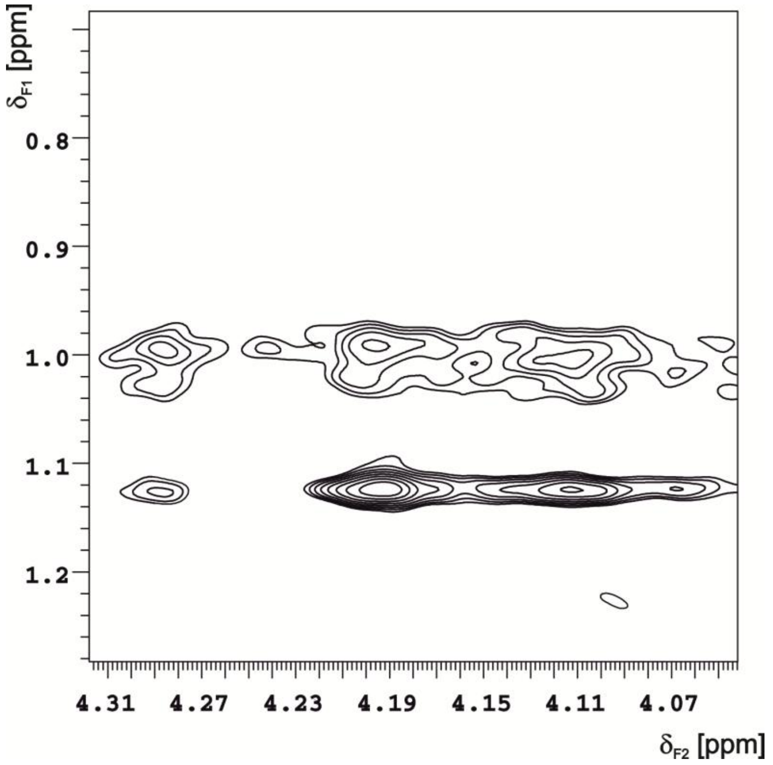

Fig. S10 The example of the cross-peaks in sample 1+3 (see experimental section). The cross peaks at 1.0 and 1.02 ppm are the intermolecular cross peaks, not assigned, of two different methyl groups 19-CH_3 due to **4** and SN38 and cross peaks at 1.12 ppm are the intramolecular cross peaks of T7-CH_3 in **1**.

Table S3. The most populated cluster energy from PBSA and GBSA analysis.

Structure	Energy [kcal/mol]	
	PBSA	GBSA
NHMe-1	-35.56 +/- 2.97	-35.68 +/- 3.03
NHMe-2	-34.43 +/- 3.19	-33.77 +/- 2.60
NHMe-3	-37.69 +/- 2.80	-34.84 +/- 2.68
NHMe-4	-34.21 +/- 3.33	-35.01 +/- 2.50

The above calculations and NOE effects in Table 4 (manuscript) point to structures 1 and 3 as the best ones. The HB also favors structure 3.

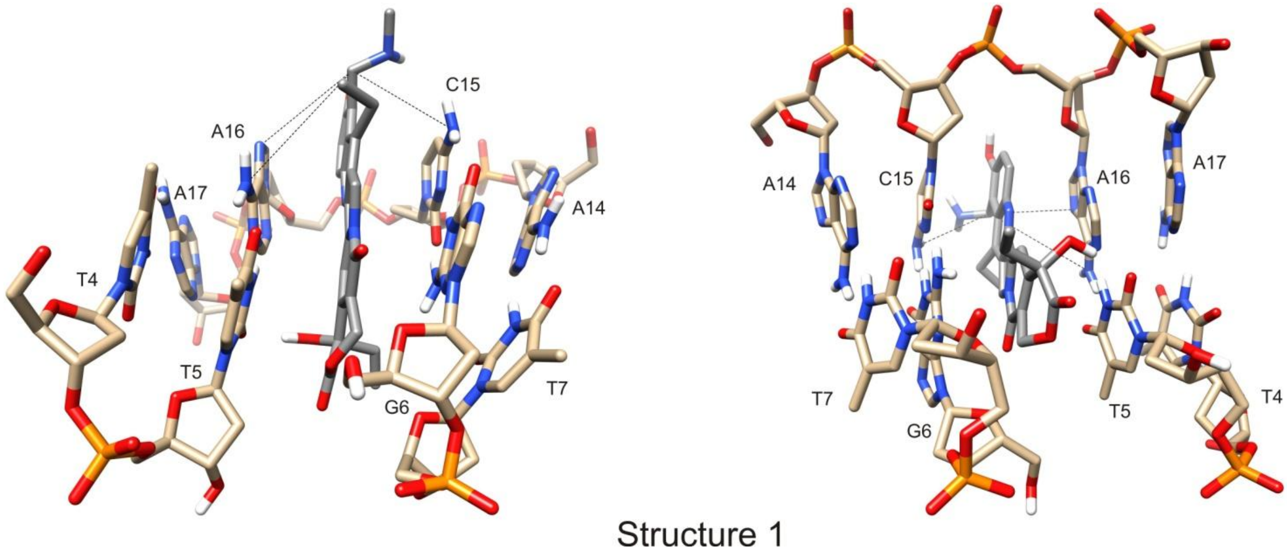

Fig. S11 The best structure from modeling showing potential sites of hybrid formation in a molecular complex **1**+**2**

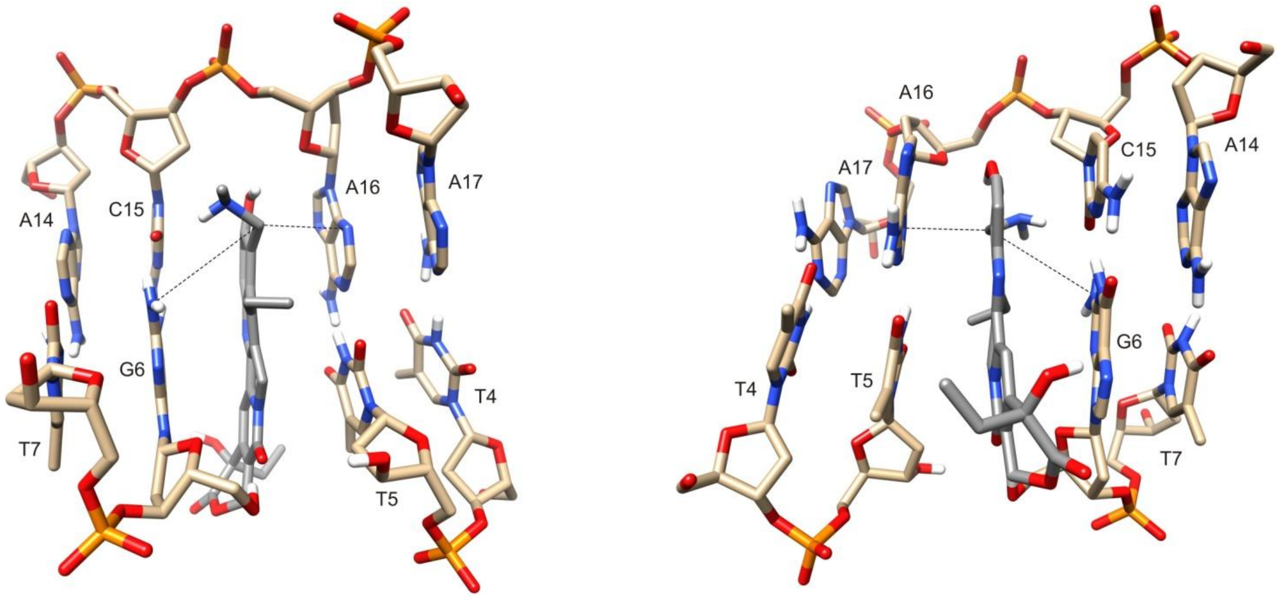

Structure 3

Fig. S12 The second best structure from modeling showing potential sites of hybrid formation in a molecular complex **1**+**2**

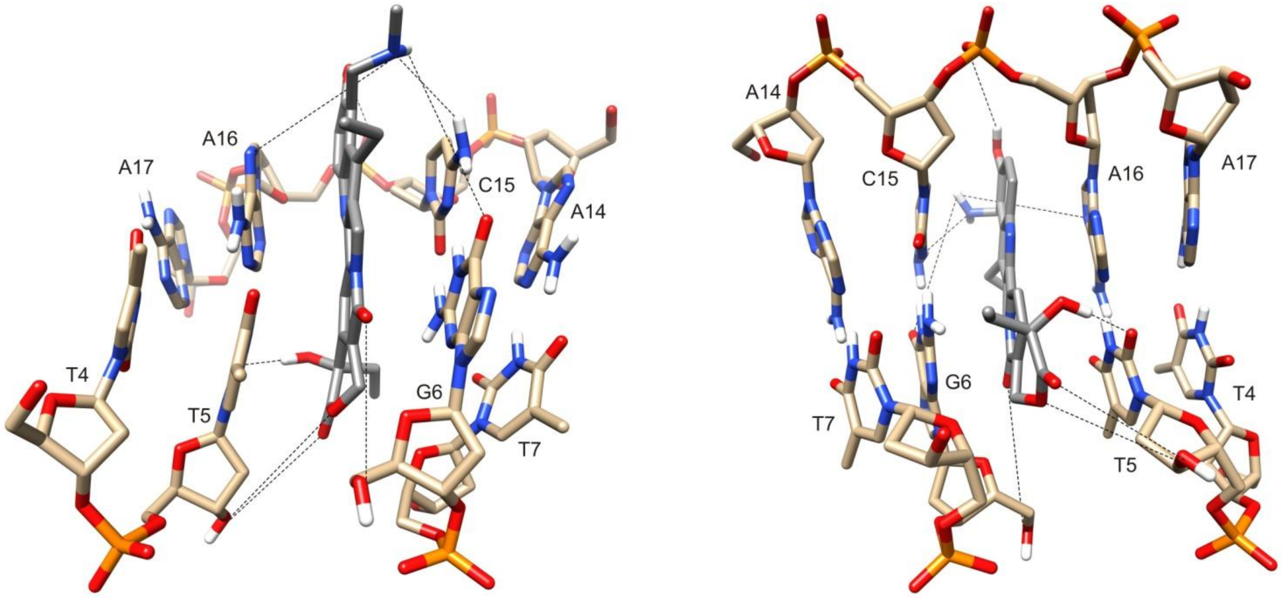

Structure 1

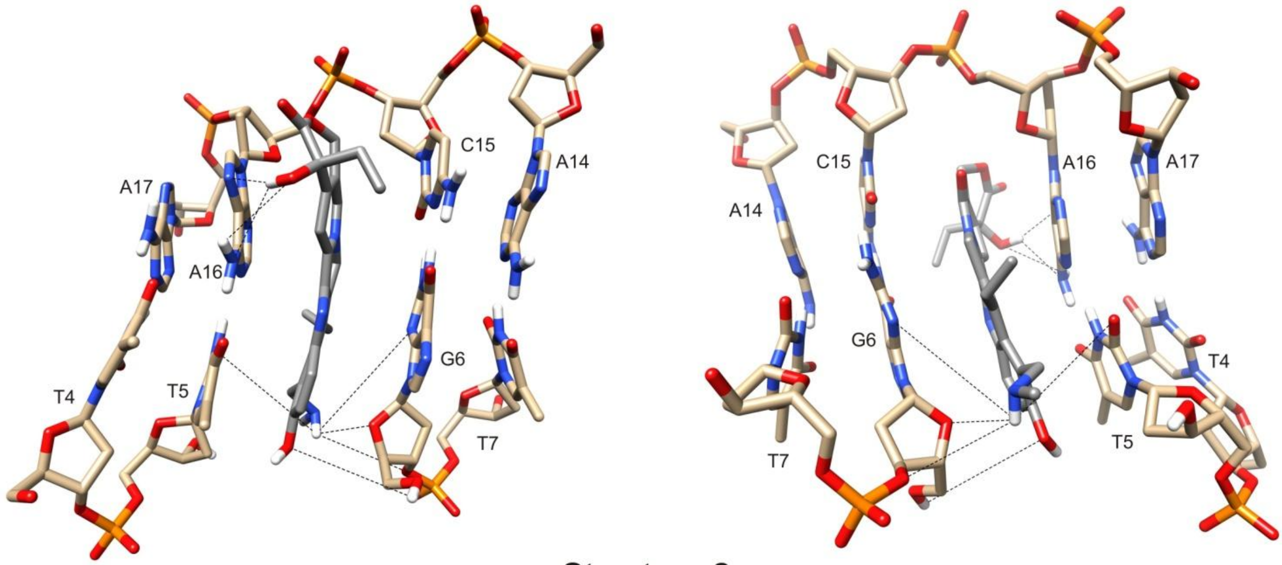

Structure 2

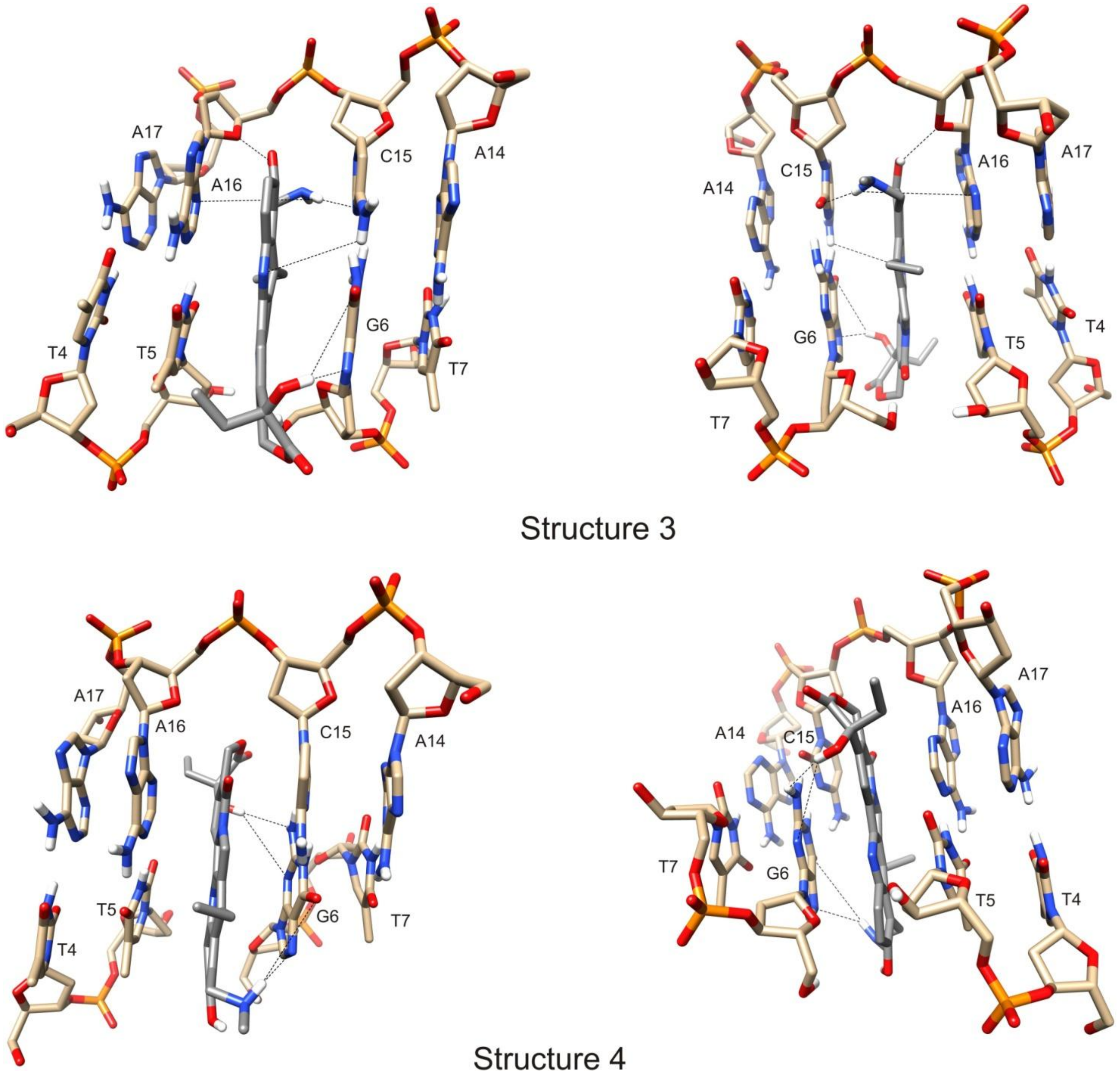

Fig. S13 The hydrogen bonding in structures best representing the most populated cluster obtained by cluster analysis in a molecular complex **1**+**2.**

Table S4 . The real hydrogen bonds in **1**+**2** complex from PM7 calculations.

Atom names		Structure 1	
DNA	CMP	HB length [Å]	population
T5-**O**2	20 -O**H**	2.08 +/- 0.29	75.5 %
G6-**H**O5'	16 >C=**O**	1.93 +/- 0.27	44.4 %
A16-**N**7	9-CH_2N**H**Me	2.26 +/- 0.10	31.5 %
T5-**H**O3'	21 >C=**O**	2.48 +/- 0.42	19.1 %
T5-**H**O3'	21 >**O**	2.85 +/- 0.28	14.0 %
A16-**O**P2	10 -O**H**	2.49 +/- 0.34	13.6 %
C15-**O**P2	10 -O**H**	2.32 +/- 0.39	7.5 %
A16-**O**5’	10 -O**H**	2.90 +/- 0.27	6.8 %
G6-**O**6	9-CH_2N**H**Me	2.49 +/- 0.28	4.1 %
		Structure 2	
A16-**N**7	20 -O**H**	2.32 +/- 0.29	63.5 %
T5-**O**2	9-CH_2N**H**Me	2.23 +/- 0.11	44.6 %
A16-**H**62	20 -**O**H	2.93 +/- 0.35	30.0 %
G6-**N**3	9-CH_2N**H**Me	2.27 +/- 0.12	20.6 %
G6-**O**4'	9-CH_2N**H**Me	2.18 +/- 0.19	18.8 %
G6-**H**O5'	10 -**O**H	1.83 +/- 0.22	16.8 %
G6-**O**3'	9-CH_2N**H**Me	2.65 +/- 0.31	10.2 %
G6-**O**4'	10 -O**H**	1.81 +/- 0.25	6.8 %
G6-**O5**'	10 -O**H**	1.78 +/- 0.29	6.2 %
		Structure 3	
C15-**O**2	9-CH_2N**H**Me	2.19 +/- 0.11	88.1 %
A16-**O**4'	10 -O**H**	2.08 +/- 0.33	78.2 %
G6-**N**7	20 -O**H**	2.21 +/- 0.14	75.3 %
G6-**H**O5'	16 >C=**O**	1.74 +/- 0.17	71.9 %
G6-**O**6	20 -O**H**	3.08 +/- 0.38	24.4 %
A16-**N**3	9-CH_2N**H**Me	2.37 +/- 0.24	3.2 %
A16-**O**5'	10 -O**H**	3.03 +/- 0.34	3.1 %
G6-**H**O5'	21 >**O**	2.27 +/- 0.36	2.9 %
		Structure 4	
G6-**N**7	9-CH_2N**H**Me	2.38 +/- 0.23	47.7 %
G6-**O**6	9-CH_2N**H**Me	2.51 +/- 0.33	29.1 %
G6-**H**21	20 -**O**H	2.54 +/- 0.46	15.1 %
G6-**N**3	20 -O**H**	2.41 +/- 0.34	13.9 %
T5-**O**4	9-CH_2N**H**Me	2.16 +/- 0.13	13.3 %
G6-**H**O5'	10 -**O**H	2.71 +/- 0.50	5.2 %

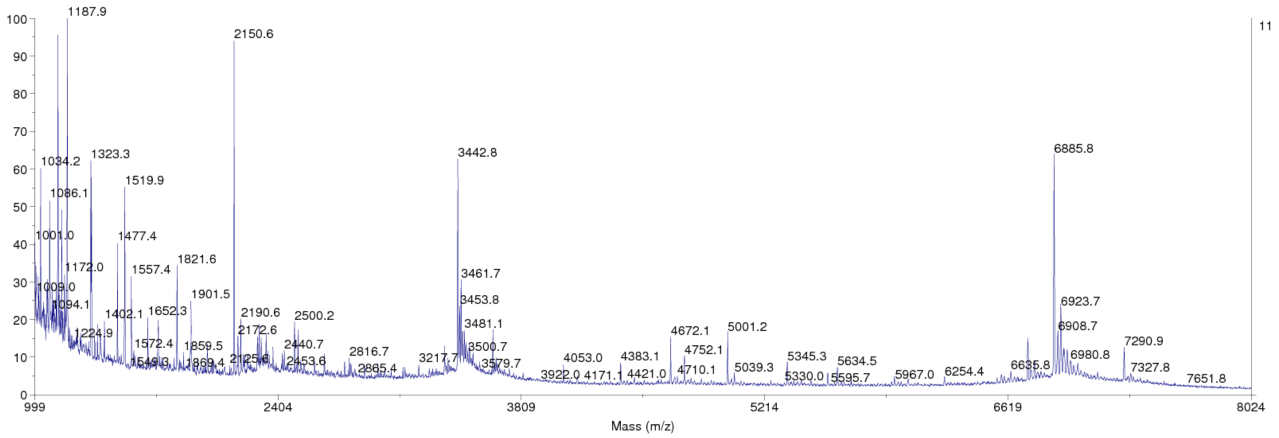

Fig. S14 The MALDI MS spectrum of lyophilized ML showing neat DNA decamer (m/z=6885.8, $[M\text{-}H]^-$) and alkylated biohybrid (m/z= 7290.9, $[M\text{-}H]^-$) with compound **2.**

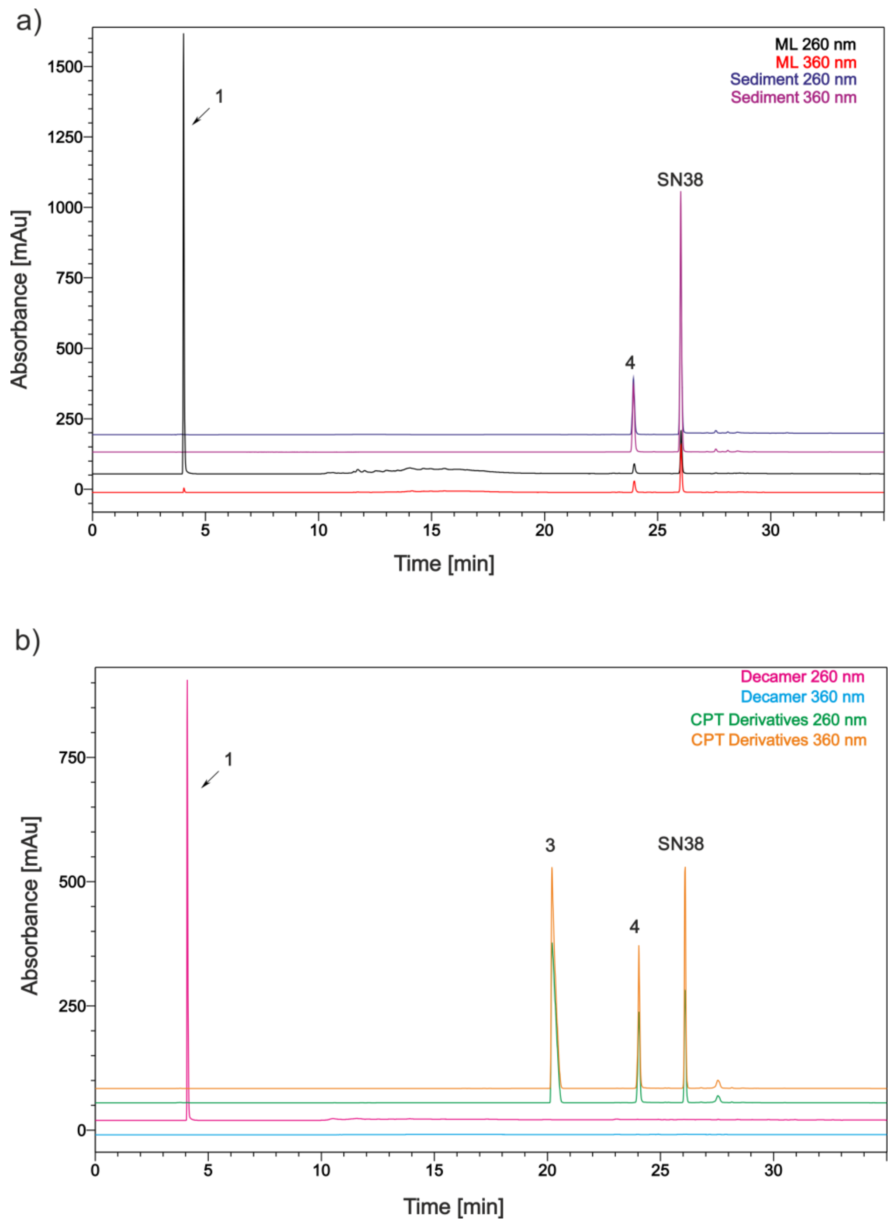

Fig. S15 Upper panel: The overlay of two HPLC runs of reaction **1** + **3**; ML in black and red and sediment in navy blue and purple. The ML run evidences compounds strongly bound to nicked DNA which partly precipitate into sediment. Lower panel: The HPLC run of reference compounds.

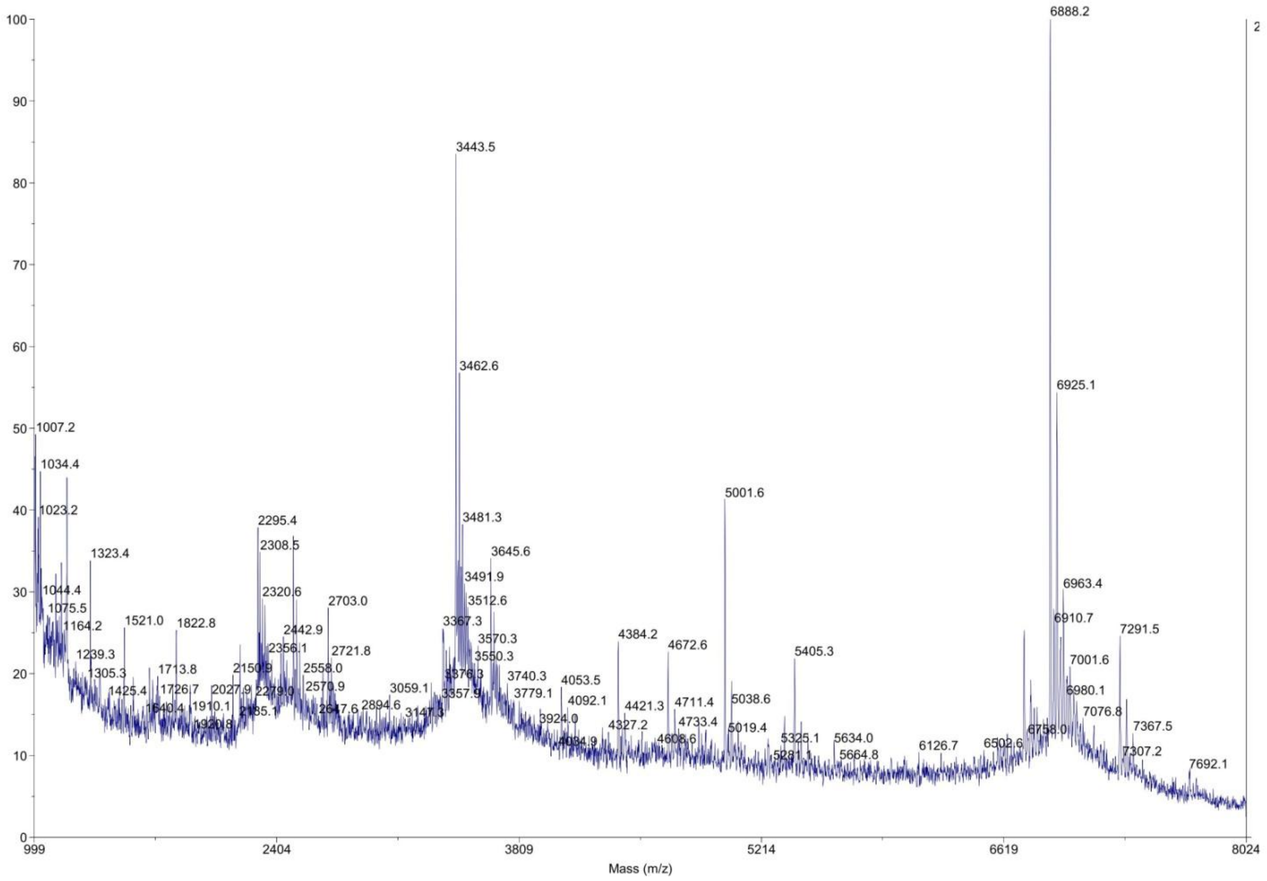

Fig. S16a The MALDI MS spectrum of lyophilized ML showing neat DNA decamer (m/z=6888.2) and alkylated biohybrid with compound **3** (m/z=7291.5) **.**

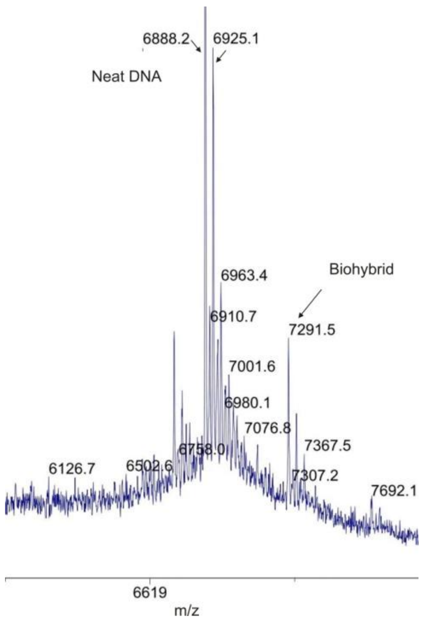

Fig. S16b The part of MALDI MS spectrum of lyophilized ML showing neat DNA decamer (m/z=6888.2, peak cut for clarity) and alkylated biohybrid with compound **3.**
